# Microbial diurnal rhythmicity in the rumen fluid impacted by feeding regimes and exogenous microbiome providing novel mechanisms regulating dynamics of the rumen microbiome

**DOI:** 10.1186/s40168-025-02134-6

**Published:** 2025-06-16

**Authors:** Yangyi Hao, Wei Wang, Mengmeng Li, Youyoung Choi, Mi Zhou, Yixin Wang, Zhijun Cao, Ya Jing Wang, Hongjian Yang, Linshu Jiang, Le Luo Guan, Shengli Li

**Affiliations:** 1 State Key Laboratory of Animal Nutrition and Feeding, Beijing Engineering Technology Research Center of Raw Milk Quality and Safety Control, College of Animal Science and Technology, China Agricultural University, Beijing, 100193 China; 2 Department of Agricultural, Food and Nutritional Science, University of Alberta, Edmonton, AB T6G 2P5 Canada; 3 Beijing Key Laboratory of Dairy Cow Nutrition, College of Animal Science and Technology, Beijing University of Agriculture, Beijing, 102206 China; 4 Faculty of Land and Food Systems, The University of British Columbia, Vancouver, BC V6 T 1Z4 Canada

**Keywords:** Rumen microbes, Circadian rhythm, Feeding regime, Rumen fluid transplantation

## Abstract

**Background:**

Diurnal oscillations have been reported on ruminal prokaryotes, but the daily rhythmicity of eukaryotes remains unknown. This study investigated diurnal oscillations of ruminal prokaryotes and eukaryotes under three different feeding managements and rumen fluid transplantation conditions, aiming to elucidate the regulatory mechanisms influencing the dynamic shifts of rumen microbiome through the daily feeding cycle.

**Results:**

Quantification and profiling of the microbiota of 288 rumen samples collected from lactating dairy cows (*n* = 12) every 6-h over 48-h feeding cycles under ad libitum, restricted feeding at daytime and nighttime, respectively, revealed the rhythmicity in the population and abundance of ruminal bacteria, archaea, and protozoa. Under restricted-feeding regimes, 61.99% bacterial genera including *Prevotella* and *Ruminococcus*, and 7.19% archaeal species including *Methanosphaera sp. ISO*3*-F*5, and 66.93% protozoa genera including *Entodinium* and *Isotricha* showed feeding-time-influenced changes in circadian rhythms. However, 4.76% bacterial genera such as *Prevotellaceae_UCG-*001, and 0.29% archaeal species such as group 12 sp. ISO4-H5 exhibited non-feeding-time affected circadian rhythm pattern shifts. Further analysis of 176 rumen fluid samples collected after rumen fluid transplantation showed the proportion of bacterial, archaeal, and protozoal taxa displayed consistent (including *Anaeroplasma* and *Fibrobacter*), inconsistent (including *Bacteroidales_UCG-*001 and *NK*4*A*214*_group*), gain (including *Prevotella* and *Succinivibrio*), and loss (including *Butyrivibrio* and *Mycoplasma*) of circadian rhythms over the 48-h to 7-day period after transplantation. Similar circadian patterns were found among feed intake, ruminal volatile fatty acid concentrations, bacterial functions such as glycolysis/gluconeogenesis, and deterministic assembly processes of bacterial communities. However, different circadian patterns (12-h shifts) were observed for rumination time, ruminal pH, ammonia nitrogen concentration, and bacterial functions such as chemotaxis, nitrogen metabolism, and deterministic assembly processes of archaeal communities. Additionally, cross-lagged effects were observed between the relative abundance of microbial taxa and rumen fermentation parameters, which could affect feed intake, rumination time, microbial population/diversity, and microbial interactions.

Video Abstract

**Conclusions:**

The classified feeding-time responsive, multi-factor responsive, consistent, and inconsistent circadian rhythm of microbial taxa underscore the driven factors behind the daily dynamics of rumen microbes, which also filled the gaps for targeting specific microbial taxa for better animal production.

**Supplementary Information:**

The online version contains supplementary material available at 10.1186/s40168-025-02134-6.

## Backgrounds

Precise manipulation of gut microbiome has been a hot topic for a healthier mankind [1] and animals [2]. With the development of the livestock industry, there has been increasing attention on the linkage among microbiota and animal health [3–6], welfare [7], and environmental sustainability [8, 9]. Ruminants digest plant feed via microbial-mediated degradation and fermentation to produce nutrients for animal growth and production, which contributes to worldwide food security; however, they are also responsible for greenhouse gas emissions from agriculture sectors [8, 10]. Recent research has shown that manipulation of the rumen microbiome through nutritional interventions could lead to lowered methane emissions [11], improved feed efficiency [2], and healthier ruminants [12]. It has been also shown that genetic selection [13], and introducing exogenous microbes (rumen fluid transplantation) [2] could be alternative approaches to changing the rumen microbiome. Among the abovementioned strategies to manipulate rumen microbiota, nutritional interventions and introducing exogenous microbes could also alter the rumen microbiota. However, none of these approaches has long term and full effects due to the complexity of the composition (bacteria, archaea, protozoa, fungi) and microbial interactions as well as host-driven individualized microbial responses to dietary/environmental changes and exogenous microbes [14]. It is necessary to identify microbes that specifically respond to different manipulation strategies, such as nutritional interventions and the introduction of exogenous microbes, which help enable more precision manipulation of the rumen microbiome.

Recent research has indicated that the diurnal oscillations overpowered the diet and individual variability in affecting the rumen bacteria and methanogens [15], suggesting that diurnal oscillations could be an important driving factor in regulating rumen microbiome composition. The circadian patterns of the microbiome have also been reported in the human gut [16] and fish skin [17]. Feeding rhythm can affect the daily patterns of gut microbiota in humans, which could further affect the expression of host biological clock genes [16], while the circadian patterns of some bacteria are associated with skin immunity in the clearance of pathogens in fish [17]. To date, most of the studies only focused on bacteria in the gut, but recent studies have reported that gut eukaryotes also play a vital role in affecting host animals such as pig growth [18] and ruminant health [19]. In the rumen, it is known that eukaryotic protozoa interact with bacteria and methanogens symbiotically [20], which can directly affect feed digestion [21] and methane emission [22]. Our previous pilot study reported that the relative abundance of the protozoal genus *Isotricha* had a circadian rhythm under both high-grain and high-forage-based diets [23]. However, there is still limited knowledge on the circadian rhythm of other ruminal microbes including archaea and protozoa, which play critical roles in feed efficiency [21] and methane emission [22]. Uncovering these circadian rhythms and the driving factors will help improve our understanding of the daily dynamics of the complex rumen microbial ecosystems and facilitate the manipulation of the microbiota in the future.

In addition, rumen microbial composition can be affected by assembly processes, which can in turn affect the outcomes of the microbial community. The assembly process refers to the mechanisms by which microbial communities are formed and maintained [24], and such process is driven by stochastic and deterministic processes [25]. The stochastic assembly processes refer that all species are ecologically equivalent, and their dynamics are affected by the species birth/death, speciation/extinction, and immigration, while the deterministic assembly processes refer that the species are controlled by the environmental filtering (e.g., pH and temperature) and biological interactions (e.g., competition and mutualisms) [25, 26]. We speculated that rumen microbiota reassembles after feeding and such assembly processes could directly affect the interactions among different microbial groups and the daily dynamics of rumen fermentation. Additionally, assembly processes are affected by the circadian rhythms of rumen microbes that can be influenced by different feeding regimes and the introduction of exogenous microbes. We further hypothesized that feeding intake and rumination could also shape the circadian rhythm of rumen microbiota. Combined with these feasible methods for precise manipulation of rumen microbiota, we assessed the circadian rhythm and assembly process of rumen microbiota under three different feeding regimes and the introduction of exogenous microbes (rumen fluid transplantation) conditions. The findings could improve our understanding of rumen microbiota temporal dynamics and facilitate the manipulation of ruminal microbiota. Additionally, the proposed study strategies could further provide a way to understand the daily dynamics of gut microbes in humans and other animals, facilitating the precise manipulation of the gut microbiome in the future.

## Methods

### Animal management

Seventeen Holstein lactating dairy cows (12 non-rumen-cannulated cows and 5 rumen-cannulated cows) with similar days in milking (213.67 ± 14.26), parity (3.25 ± 0.72), and body weight (765.67 ± 47.95 kg) were selected for the experiment. All cows were kept in a free-stall barn and milked twice a day (08:00 and 20:00) following the herd standard operation protocol at the Beijing Nainiu Center Farm (Yanqing, Beijing, China).

### Experimental design, samples, and phenotype data collection

**Fig. 1:**
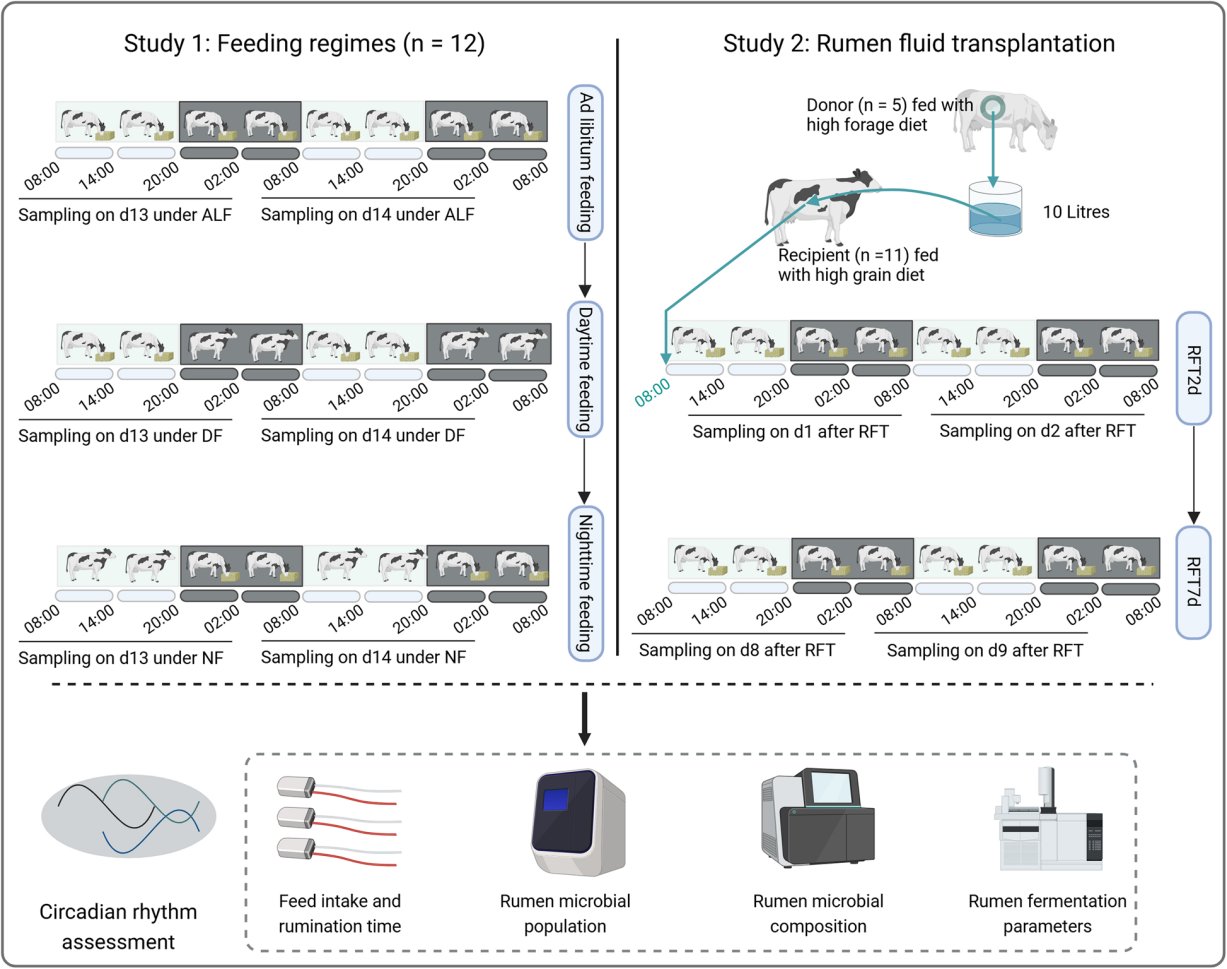
Experimental design illustration. ALF, ad libitum feeding; DF, daytime feeding; NF, nighttime feeding. RFT2d, 48 h after rumen fluid transplantation. RFT7d, 7 days after rumen fluid transplantation

This work involved two animal studies, and the sketch of the experiments and sample collections were shown in Fig. 1. Study 1 was the different feeding regimes study: 12 non-rumen-cannulated cows were enrolled in the feeding trials and they were fed with a high-grain diet (Suppl File S1) under three different feeding regimes: ad libitum feeding (ALF: feed available for the whole day), daytime feeding (DF; feed available from 08:00 to 20:00), and nighttime feeding (NF; feed available from 20:00 to 08:00), respectively. For each feeding regime, the treatment lasted 14 days, followed by a 7-day interval prior to switching to a different feeding regime, with a total of 56 days for the entire study. Rumen fluid was collected every 6 h (in total of 8 times) during the last 2 days of each feeding regime, at 08:00, 14:00, 20:00, and 02:00, for each day, respectively. At each sampling, a total of 50 mL rumen fluid was collected per cow via oral gastric tube (Ancitech, Winnipeg, MB, Canada) and separated into two parts, one part was snap-frozen with liquid nitrogen for later DNA extraction, and the other part was stored at − 20 ℃ for fermentation parameters measurement. Study 2 was the rumen fluid transplantation (RFT) study. For the RFT trial, five rumen-cannulated dairy cows were used as donors and were fed a high-forage diet for 3 weeks before RFT. The recipients were eleven non-rumen-cannulated cows, which were fed a high-grain diet. The ingredients and chemical composition of the high-forage and high-grain diets were presented in Suppl File S1. Briefly, the rumen fluid was collected from the donor cows before morning feeding (08:00) and pooled together and then immediately orally drenched 10 L to each of the recipients. Previous study reported that the volume of transplantation of rumen fluid ranged between 10 and 11.4 L per oral dose, and such an amount was sufficient to alter the ruminal microbial community in adult cattle [27]. To compare the immediate and short-term effects of rumen fluid transplantation on ruminal microbiota diurnal oscillations, we collected the rumen fluid within 48 h after transplantation (termed RFT2d group), and 7 days after rumen fluid transplantation (termed RFT7d group), respectively. Specifically, rumen fluid was collected at 8 time points within 2 days immediately after RFT (RFT2d) and 7 days after RFT completion (RFT7d) (Fig. 1).

Cows’ feed intake activity was recorded by the Roughage Intake Control System (RFID, Zhenghong Company, Shanghai, China). Rumination activity was monitored by the Neck-Mounted Accelerometer-Equipped Collars (Merck & Co., Inc., Rahway, NJ, USA). The feed intake and rumination time data were recorded throughout all the experimental periods.

### Rumen fermentation parameter measurement

Rumen fluid pH was measured immediately after sample collection with the pH electrode (model pH B-4; Shanghai Chemical, Shanghai, China). Ammonia nitrogen (NH_3_-N) concentration was measured using the phenol-sodium hypochlorite colorimetry method on a spectrophotometer (721-G, INESA analytical instrument Co., LTD, Shanghai, China) described by Broderick and Kang [28]. The volatile fatty acid (VFA) was measured using gas chromatography (6890 N; Agilent Technologies, Avondale, PA, USA) with a capillary column (0.32 mm × 0.50 mm film thickness) following the methods described by Cao et al. [29].

### DNA extraction and amplicon sequencing

The total DNA was extracted from 1-mL rumen fluid using the QIAmp Stool Mini Kit (Qiagen, Germany) according to the manufacturer’s instructions. Quality and quantity of the DNA were assessed based on absorbance at 260 and 280 nm using an ND-1000 spectrophotometer (Nanodrop Technologies, Wilmington, USA). Thirty nanograms of DNA was used for amplicon generation using group-specific primers and subjected to amplicon sequencing. For bacteria, the primers were Ba9f (5’-GAGTTTGATCMTGGCTCAG-3’) and Ba515Rmod1 (5’-CCGCGGCKGCTGGCAC-3’); for archaea, the primers were Ar915aF (5’-AGGAATTGGCGGGGGAGCAC-3’) and Ar1386R (5’-GCGGTGTGTGCAAGGAGC-3’); for protozoa, the primers were Reg841F (5’-GACTAGGGATTGGAGTGG-3’) and Reg1302R (5’-AATTGCAAAGATCTATCCC-3’) [30]. The polymerase chain reaction (PCR) program included an initial denaturation at 95 ℃ for 3 min, followed by 27 (bacteria), 35 (archaea), and 35 (protozoa) cycles of 95 ℃ for 30 s, annealing at 55 ℃ for 30 s, elongation at 72 ℃ for 45 s, and last a final elongation at 72 ℃ for 10 min. Negative control with sterile water was included during DNA extraction and subjected to qPCR during amplicon preparation to check for contamination, using the same primers and conditions as for the samples. The obtained DNA amplicons were subjected to library construction and sequencing using Illumina PE Miseq (300 bp pair-end).

### Sequencing data analysis

The raw sequence data were assigned to each sample based on the corresponding barcode and were processed using QIIME2 (Version 2022.2) [31]. Quality control, denoising, removal of chimeric sequences, and generation of amplicon sequencing variants (ASVs) were performed using the QIIME2 plugin DADA2 [32]. Taxonomy analysis was performed with the feature classifier command in QIIME2 by aligning the ASVs against the SILVA database (Version 138 [33]) for bacteria and protozoa, and the RIM-DB database [34] for archaea. The adequacy of sequencing depth was evaluated by Good’s coverage index. Alpha diversity (Shannon: richness and Chao1: evenness) was calculated using the scripts implemented in QIIME2 with the depth of 5781, 1412, and 2287 ASVs for bacteria, archaea, and protozoa, respectively. The functional profiles of bacteria and archaea were predicted using the “q2-picrust2” plugin in QIIME2 [35].

### Quantify the ruminal microbiota population

The quantitative polymerase chain reaction (qPCR) was performed to quantify the population of ruminal bacteria, archaea, and protozoa. Three pairs of universal primers were used for qPCR: total bacteria (U2-F: 5’-ACTCCTACGGGAGGCAG-3’; U2-R: 5’-GACTACCAGGGTATCTAATCC-3’) [36], total archaea (uniMet1-F: 5’-CCGGAGATGGAACCTGAGAC-3’; uniMet1-R: 5’-CGGTCTTGCCCAGCTCTTATTC-3’) [37], and total protozoa (SSU-316F: 5’-GCTTTCGWTGGTAGTGTATT-3’; SSU-539R: 5’-CTTGCCCTCYAATCGTWCT-3’) [38]. The qPCR was conducted using SYBR Green chemistry (Fast SYBR Green Master Mix; Applied Biosystems) in the StepOnePlus Real-time PCR System (Applied Biosystems) with a holding stage, a fast cycle, and then a melt curve section. The PCR program was as follows: for bacteria, the holding stage started at 95 ℃ for 5 min, followed by 40 cycles at 95 ℃ for 20 s and 60 ℃ for 30 s; for archaea, the holding stage started at 95 ℃ for 20 s, followed by 40 cycles at 95 ℃ for 3 s and 62 ℃ for 30 s; for protozoa, the starting temperature was 95 ℃ 20 s, followed by 40 cycles at 95 ℃ for 3 s and 60 ℃ for 30 s. For melting curve detection, all started at 95 ℃ for 15 s, followed by 60 ℃ for 1 min, and then, the temperature was increased by 0.3 ℃ every 20 s from 60 ℃ to 95 ℃, then kept at 95 ℃ for 15 s. Standard curves were made using serial dilutions of purified plasmid DNA, and the initial concentration was 3.42 × 10^10^ mol/µl, 2.77 × 10^10^ mol/µl, and 2.78 × 10^10^ mol/µl for bacteria, archaea, and protozoa, respectively. The copy numbers of each standard curve were calculated based on the formula (*N*_L_ × *A* × 10^−9^)/(660 × *n*), where *N*_L_ is the Avogadro constant (6.02 × 10^23^ molecules per mol), *A* is the molecular weight of the molecule in standard, and *n* is the length of the amplicon (bp). The total copy numbers of bacteria, archaea, and protozoa per milliliter rumen fluid were calculated using the formula (*M*_*Q*_ × *C* × *V*_*D*_)/(*S* × *V*), where *M*_*Q*_ is the quantitative mean of the copy number, *C* is the DNA concentration of each sample, *V*_*D*_ is the eluted volume of extracted DNA, *S* is the DNA amount (ng) subjected to qPCR analysis, and *V* is the rumen fluid volume subjected to DNA extraction.

### Assessment of microbial community assembly process

The infer community assembly mechanisms by phylogenetic bin-based null model analysis (iCAMP) framework were used to assess the rumen microbial community assembly processes [39]. The iCAMP could differentiate the relative importance of five assembly processes including heterogeneous selection (HeS), homogeneous selection (HoS), homogenizing dispersal (HD), dispersal limitation (DL), and drift and others (DR) at both the whole community and bin levels [40]. The HeS and HoS constituted deterministic processes, and the HD, DL, and DR contributed to stochastic processes in iCAMP. The term “bin” meant a group of ASVs that were closely phylogenetically related [40]. For the construction of ASV bins, the highest relative abundance of ASV was designated as the centroid taxon of the first bin. All taxa with a distance to the centroid taxon less than the phylogenetic signal threshold of 0.2 were assigned to that bin until the bin size was 24 (the bin contains 24 ASVs) [39]. The next bin was generated from the rest of the taxa in the same way.

### Rumen microbial network construction and microbial taxa niche breadth calculation

To uncover the diurnal oscillations of interaction among microbial taxa, networks among the detected bacteria genera, archaea species, and protozoa genera were analyzed using the SparCC program [41] implemented with R package “SpiecEasi” (version: 1.1.2) with bootstrapping (100 times) to get the correlation significance *P* value [42] in R studio. Only correlations with coefficients > 0.3 or < − 0.3 and *P* < 0.05 were selected for the network topology and network node feature analysis. Network topology, network node feature, and modularity were calculated in R package “igraph” (version: 1.3.5). Based on the within-module connectivity (Zi) and among-module connectivity (Pi), taxa could be assigned to specialists and generalists. Specialists included peripheral taxa (Zi < 2.5, Pi < 0.62), which were connected at least 60% links within the module [43]; generalists referred to the microbial taxa that were highly connected with others both within and among module (Network hubs: Zi > 2.5, Pi > 0.62), within a module (module hubs: Zi > 2.5, Pi < 0.62), and among the different module (connectors: Zi < 2.5, Pi > 0.62) [43].

Levins’ niche breadth index (${B}_{j}$) [44] was estimated to uncover the capacity of the rumen microbiota to survive in the environment, which further contributed to the diurnal oscillations of microbial taxa. The ${B}_{j}$ compute algorithm is as follows:

$${B}_{j}=1/{\sum }_{i=1}^{N}{P}_{ij}^{2}$$

${B}_{j}$ was the niche breadth of taxon $j$ in a metacommunity. $N$ was the total number of communities in each metacommunity. ${P}_{ij}$ was the relative abundance of taxon $j$ in community $i$. A higher ${B}_{j}$ indicated a wider habitat niche breadth, which was more metabolically flexible [45].

### Identification of the ruminal microbiota circadian rhythm pattern features

We use total gene copy numbers multiplied by relative abundance to obtain the estimated absolute abundance of each microbial taxon. The relative abundance of microbial taxa, estimated absolute abundance of microbial taxa, and the relative abundance of the predicted pathways were fit to the cosinor model with the “Circacompare” R package (version: 0.1.1) to identify if they were circadian rhythms significantly and their circadian patterns [46]. In the cosinor model, mesor, amplitude, and peak time were calculated to represent the circadian patterns of microbial taxa. The mesor represented the rhythm-adjusted mean value of the measurement [46], a higher mesor indicated a higher value of midline estimating statistics of rhythms. Amplitude represented the extent of predictable change within a cycle [46], a higher amplitude indicated the measurement was more fluctuant within a day. Peak time represented the time in the peak of rhythm [46], which indicated the time that the measurement arrived at its highest value within a feeding cycle (24 h). In order to know the difference in the circadian rhythm patterns under different feeding-restriction regimes or the immediate- and short-term effects of exogenous microbiota transplantation, the circadian patterns of microbial taxa between DF and NF groups, as well as RFT2d and RFT7d groups were further compared by the “circacompare_mixed” function in “Circacompare” R package.

### Exploration of the cross-lagged relationships between microbial taxa and rumen fermentation parameters, as well as feed intake and rumination

In order to understand the rumen fermentation efficiency, the estimated methane emission (ECH_4_) was calculated as the following formula [47], which represented the molar amount of methane molecules generated when one molar of total TVFA was produced:

$${ECH}_{4}= 2*Acetate \%-Propionate \%+2* Butyrate \%-Valerate \%$$

The cross-lagged panel model (CLPM) was used to assess the cross-lagged correlations (a type of relationship between two variables where changes in one variable at an earlier time point affected changes in the other variable at a later time point, and vice versa) between feed intake, rumination, fermentation parameters, and microbial taxa throughout the day [48]. Before performing the CLPM analysis, the samples were regrouped based on after-feeding at 6, 12, 18, and 24 h and averaged among ALF, DF, NF, RFT2d, and RFT7d groups. The hypothesized conceptual model for CLPM was presented in Fig. S1, and the CLPM analysis was performed with the “lavaan” package (version: 0.6–18). The model fit was evaluated using the *P* value, the comparative fit index, the root-mean-square error of approximation, and the standardized root-mean-square residual. Only the path coefficient > 0.30 or < − 0.30 was considered meaningful in our study.

### The cause-and-effect relationship among feed intake, as well as rumination, microbiota, and ruminal fermentation parameters

Before exploring the cause-and-effect relationship, the correlation between microbial diversity and feed intake, as well as rumination, microbial populations, and ruminal fermentation parameters was evaluated with the linear mixed model (LMM) [49] using “lme4” (version: 1.1.31) R package, in which sampling cows were termed as random effects. Due to the consecutive 2 days of sampling being auto-correlated, a nested autoregressive model was added to account for temporally correlated errors within the LMM. The *P* value from the LMM was assessed by the Wald type II *χ* ^2^ tests using the “car” R package (version: 3.1.1). The regression coefficients in the LMM represented the relationship between microbiota and feed intake, as well as the rumination and fermentation parameters. For the microbial Shannon and Chao1 indices, their regression coefficients in LMM with a higher absolute value were chosen for cause-and-effect relationship analysis.

Partial least squares path modeling (PLS-PM) was used to explore the cause-and-effect relationships among the rumen microbial diversity, microbial population, fermentation parameters, cows’ feed intake, and rumination time [50]. We first considered a hypothesized conceptual model (Fig. S2) that included all reasonable pathways. The path coefficients and the coefficients of their *P*-value were validated using 1000 bootstraps. Path coefficients represented the direction and strength of the linear relationships among the different variables (direct effects). PLS-PM model is performed in R Studio with the R package “plspm” (version: 0.5.0), and the model reliability was evaluated using the goodness of fit statistics.

### Statistical analysis

The daily patterns of feed intake, rumination time, microbial population, microbial Chao1, and Shannon indices, as well as ruminal fermentation parameters, were evaluated using the cosinor model with the “Circacompare” R package. This model assessed whether these variables followed a significant circadian rhythm and determined their circadian patterns (mesor: rhythm-adjusted mean value, calculated as the average value of the oscillating data; amplitude: extent of change within a cycle, calculated with the distance between the mesor to the peak value in the cosinor model; and peak time: the time when the measurement reaches its highest value within a 24-h feeding cycle) [46]. The significance of the relative importance of ecological processes between different feeding regimes or between the RFT2d and RFT7d groups was calculated by permutational *t* test (1000 times). The circadian rhythm of the microbial community assembly process was evaluated with the “circa_single” function of the “Circacompare” R package. In addition, we averaged the dataset of all experimental cows at each sampling point, and the correlation between the assembly process and feed intake, rumination, and rumen fermentation parameters under each group was assessed using Spearman rank correlation in SPSS (version 28). The Mantel test was conducted using the “vegan” package (version: 2.6.4) to uncover the diurnal oscillations of overall correlation among the bacterial, archaeal, and protozoal communities. Finally, the association between node features in the networks and their circadian rhythm parameters, as well as niche breadth, was measured with Spearman rank correlation in SPSS. The *P* < 0.05 indicated a significant difference for all the analyses. The tendency was considered 0.05 < *P* ≤ 0.10.

## Results

### Microbial profiling data overview

A total of 31,039,601 (65,346 ± 18,635, mean ± standard deviation), 8,952,823 (18,848 ± 7,501), and 11,866,578 (24,982 ± 21,082) raw reads were obtained for bacteria, archaea, and protozoa, respectively, from 475 rumen fluid samples collected from: (1) every 6 h for the period of 48 h under three different feeding regimes and (2) every 6 h for the period of 48 h immediately after rumen fluid transplantation (RFT2d) and 7 days after rumen fluid transplantation (RFT7d) (Fig. 1). After quality control, 7,416,917 (15,614 ± 5,567), 2,393,327 (5,038 ± 2,465), and 4,771,322 (10,044 ± 11,408) of non-chimeric reads remained and were assigned to 51,943, 876, and 1,140 ASVs for bacteria, archaea, and protozoa, respectively. In total, 196 bacterial genera, 14 archaeal species, and 6 protozoal genera were identified (Suppl File S2: Sheet 1).

### The circadian rhythm of rumen microbial diversity and population

Bacterial alpha diversity (Chao1 and Shannon indices) showed significant circadian rhythms (*P* < 0.05) in ALF, DF, NF, and RFT7d groups (Fig. S3 A1-3, A5, B1-3, and B5), while no circadian rhythm of them was detected in the RFT2d group (Fig. S3 A4 and B4). Archaeal Chao1 index showed a significant circadian rhythm (*P* < 0.05) in the ALF and RFT7d groups (Fig. S3 A6 and A10), and Shannon index showed a significant circadian rhythm (*P* < 0.05) in the DF, RFT2d, and RFT7d groups (Fig. S3 B7, B9, and B10). Protozoal Chao1 and Shannon indices showed significant circadian rhythms (*P* < 0.05) in the DF and RFT2d groups (Fig. S3 A12, A14, B12, and B14). The bacterial population estimated by 16S rRNA gene copy numbers showed a significant circadian rhythm (*P* < 0.05) in ALF, DF, NF, RFT2d, and RFT7d groups (Fig. S4 A1-5). The archaeal population showed a significant circadian rhythm (*P* < 0.05) in ALF, DF, and RFT7d groups (Fig. S4 B1, B2, and B5), while the protozoal population only showed a significant circadian rhythm in DF, NF, and RFT2d groups (*P* < 0.05) (Fig. S4 C2-4).

### The circadian rhythm of rumen microbial taxa under different feeding regimes

**Fig. 2:**
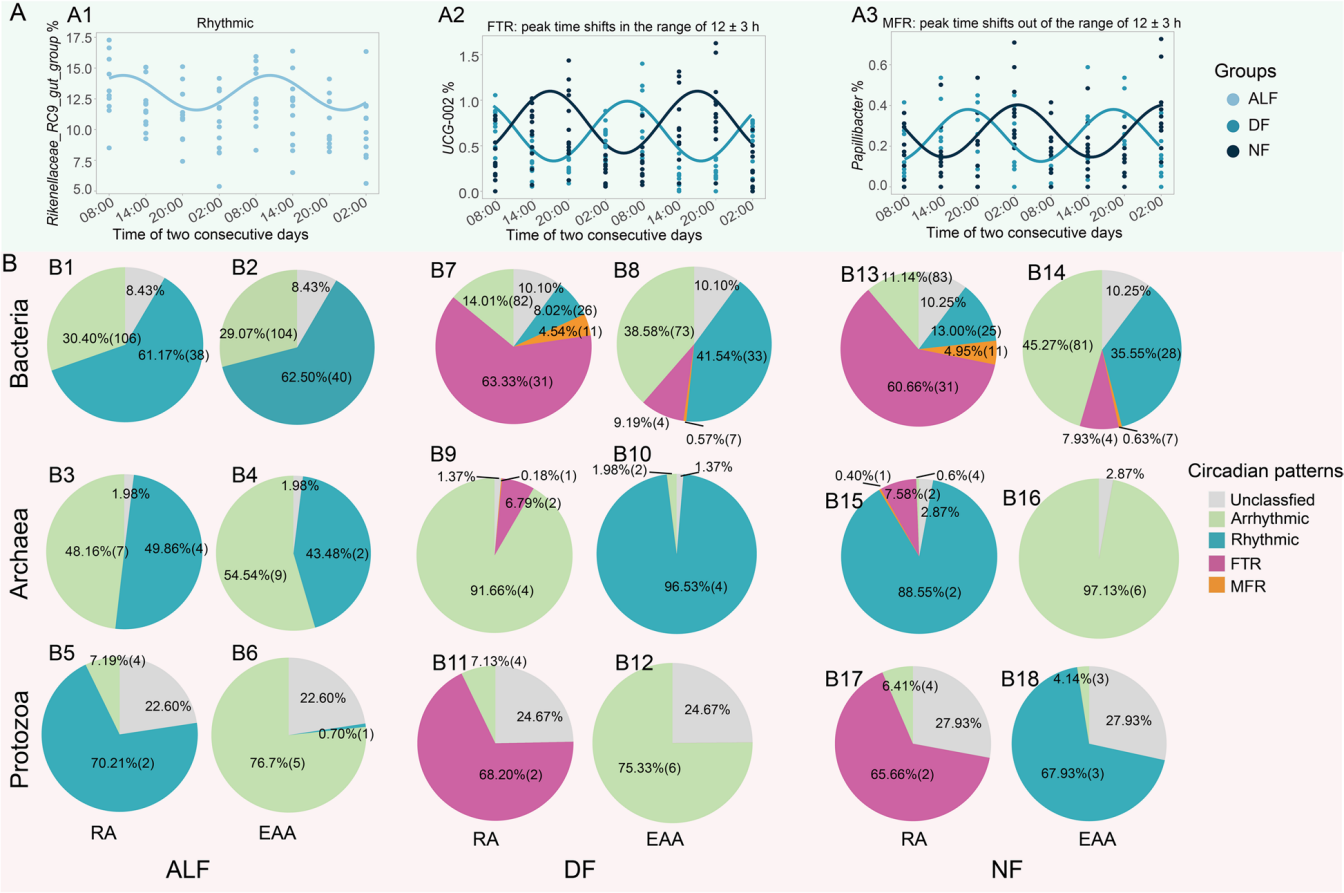
Figure legend (**A**) and the proportion of circadian rhythm patterns of microbial taxa (**B**) under different feeding regimes. ALF, ad libitum feeding; DF, daytime feeding; NF, nighttime feeding. Arrhythmic taxa, the *P* value > 0.05 based on Circacompare analysis. Rhythmic taxa, the *P* value < 0.05 based on Circacompare analysis. FTR, feeding time responsive taxa, the taxa in both DF and NF groups showed significantly circadian rhythm (*P* < 0.05), and the difference in the peak of rhythm between the two groups was in the range of 12 h ± 3. MFR, multi-factor responsive taxa, the taxa in both DF and NF groups showed significantly circadian rhythm (*P* < 0.05), and the difference in the peak of rhythm between the two groups was out of the range of 12 h ± 3. RA, relative abundance; EAA, estimated absolute abundance

Under different feeding regimes, the circadian rhythm patterns of microbial taxa’s relative abundance were classified. Firstly, the relative abundances of some taxa showed rhythmic patterns (e.g., genus *Rikenellaceae_RC*9*_gut_group* in Fig. 2A1), while those of some taxa exhibited arrhythmic under ad libitum feeding conditions. For these rhythmic taxa, some of them showed a consistent circadian pattern shift with feeding time shift (the peak time shift in the range of 12 ± 3 h) between DF and NF regimes, suggesting the circadian patterns of the relative abundance of these taxa were driven by the feeding time, which were classified as feeding-time responsive (FTR) taxa (e.g., genus *UCG* −002 in Fig. 2A2). Some of rhythmic taxa showed circadian patterns shift (the peak time shift out the range of 12 ± 3 h) inconsistent with feeding time shift between DF and NF regimes, suggesting that in addition to feeding time, there were still some unknown factors affecting the circadian rhythm patterns of these taxa, which were classified as multi-factor responsive (MFR) taxa (e.g., genus *Papillibacter* in Fig. 2A3). Specifically, under the ALF regime, 38 bacterial genera (accounting for 61.17% of total bacterial reads, same as for archaea and protozoa; Fig. 2B1), 4 archaeal species (49.86%; Fig. 2B3), and 2 protozoal genera (70.21%; Fig. 2B5) had a significant circadian rhythm in terms of their relative abundance (Fig. 2). In addition, 40 bacterial genera (62.50%; Fig. 2B2), 2 archaeal species (43.48%; Fig. 2B4), and 1 protozoal genera (0.70%; Fig. 2B6) were rhythmic based on their estimated absolute abundance (calculated based on relative abundance multiply by respective 16S or 18S rRNA genes copy numbers). Within the feeding-restricted regimes, the relative abundances of the following taxa were classified as FTR: 31 bacterial genera (DF: 63.33%; Fig. 2B7 and NF: 60.66%; Fig. 2B13) such as *Prevotella* and *Ruminococcus*, 2 archaeal species Group8.sp* WGK*1 and *Methanosphaera *sp. ISO3-F5 (DF: 6.79%; Fig. 2B9 and NF: 7.58%; Fig. 2B15), and 2 protozoal genera *Entodinium* and *Isotricha* (DF: 68.20%; Fig. 2B11 and NF: 65.66%; Fig. 2B17). Furthermore, 11 bacterial genera (DF: 4.54%; Fig. 2B7 and NF: 4.95%; Fig. 2B13) such as *Prevotellaceae_UCG-*001 and *Anaeroplasma*, 1 archaeal species group 12 sp.ISO4-H5 (DF: 0.18%; Fig. 2B9 and NF: 0.40%; Fig. 2B15), and no protozoal taxa were categorized as MFR taxa. Based on the estimated absolute abundance, 4 bacterial genera (DF: 9.19%; Fig. 2B8 and NF: 7.93%; Fig. 2B14) and 7 bacterial genera (DF: 0.57%; Fig. 2B8 and NF: 0.63%; Fig. 2B14) were classified as FTR taxa and MFR taxa, respectively, while no archaeal or protozoal taxa were categorized as either FTR taxa or MFR taxa. All the detailed circadian patterns of the microbial taxa under different feeding regimes were listed in the Suppl File S2 (Sheet 2, ALF; and Sheet 3, restricted feeding regimes).

**Fig. 3:**
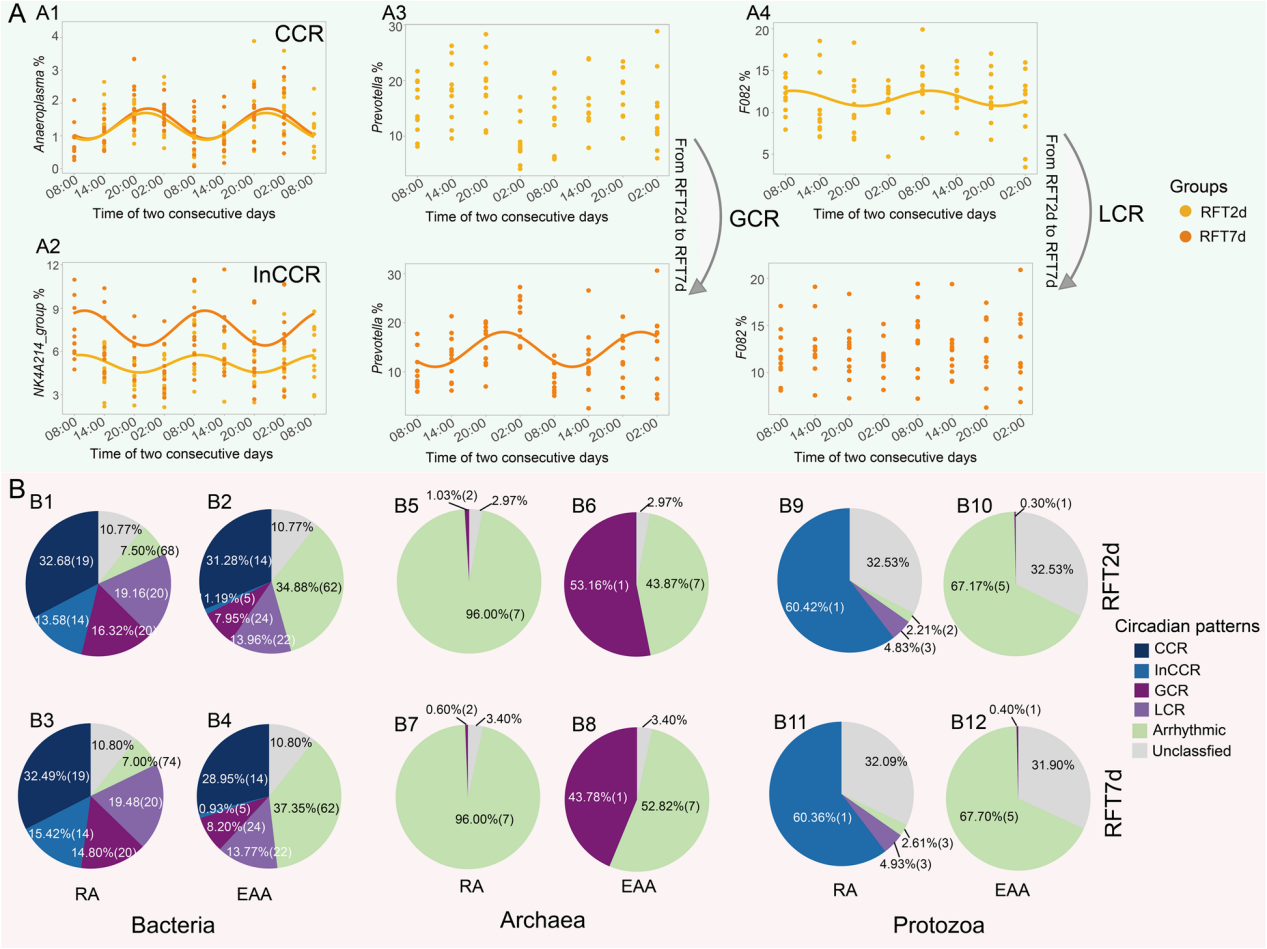
Figure legend (**A**) and the proportion of different circadian rhythm patterns of microbial taxa (**B**) in RFT2d and RFT7d groups. RFT2d, 48 h after rumen fluid transplantation. RFT7d, 7 days after rumen fluid transplantation. CCR, consistent circadian rhythm taxa, the *P* value of their circadian rhythm < 0.05 in both RFT2d and RFT7d groups, and the circadian rhythm pattern parameters had no difference between the two groups. InCCR, inconsistent circadian rhythm taxa: the *P* value of their circadian rhythm < 0.05 in both RFT2d and RFT7d groups, and the circadian rhythm pattern parameters had a significant difference between the two groups. GCR, gain circadian rhythm taxa, the *P* value of their circadian rhythm > 0.05 in the RFT2d group, and it changed to < 0.05 in the RFT7d group. LCR, loss of circadian rhythm taxa, the *P* value of their circadian rhythm < 0.05 in the RFT2d group, and it changed to > 0.05 in the RFT7d group. RA, relative abundance; EAA, estimated absolute abundance

### The circadian rhythm of rumen microbial taxa after the introduction of exogenous rumen microbes

We first compared the donor’s rumen fluid microbiota with recipients, and there was a significant difference between them based on analysis of similarities with *β* diversity (weighted UniFrac distance) (Fig. S5). The analysis of the circadian rhythm of rumen microbiota after rumen fluid transplantation showed that some taxa had a significant circadian rhythm, and their circadian parameters had no difference between RFT2d and RFT7d groups, which were termed as consistent circadian rhythm (CCR) taxa (e.g., genus *Anaeroplasma* in Fig. 3A1). In the meantime, the taxa had a significant circadian rhythm, and their circadian patterns had a significant difference between RFT2d and RFT7d groups, which were classified as inconsistent circadian rhythm (InCCR) taxa (e.g., genus *NK*4*A*214*_group* in Fig. 3A2). Additionally, some microbial taxa were found to lose their circadian rhythm within 48 h after rumen fluid transplantation but gained the circadian rhythm 7 days after rumen fluid transplantation, which was classified as gained circadian rhythm (GCR) taxa (e.g., genus *Prevotella* in Fig. 3A3). Those who showed circadian rhythm within 48 h after transplantation but lost it 7 days after rumen fluid transplantation were categorized into the loss of circadian rhythm (LCR) taxa (e.g., genus *F*082 in Fig. 3A4). In the relative abundance dataset, nineteen bacterial genera (RFT2d: 32.68%; Fig. 3B1 and RFT7d: 32.49%; Fig. 3B3) including *Ruminococcus* and *Fibrobacter* were classified as CCR taxa, while no archaeal and protozoal taxa were identified as CCR. Fourteen bacterial genera (RFT2d: 13.58%; Fig. 3B1 and RFT7d: 15.42%; Fig. 3B3) including *Bacteroidales_UCG-*001 and *UCG-*004, and one protozoal genera *Entodinium* (RFT2d: 60.42%; Fig. 3B9 and RFT7d: 60.36%; Fig. 3B11) was classified as InCCR. Twenty (RFT2d: 16.32%; Fig. 3B1 and RFT7d: 14.80%; Fig. 3B3) bacterial genera including *Prevotella* and *Succinivibrio*; 2 (RFT2d: 1.03%; Fig. 3B5 and RFT7d: 0.60%; Fig. 3B7) archaeal species group 12 sp. ISO4-*H*5 and group 9 sp. ISO4-G1 were classified as GCR. Additionally, 20 (RFT2d: 19.16%; Fig. 3B1 and RFT7d: 19.48%; Fig. 3B3) bacterial genera including *Butyrivibrio* and *Mycoplasma*, and 3 (RFT2d: 4.83%; Fig. 3B9 and RFT7d: 4.93%; Fig. 3B11) protozoal genera including *Dasytricha*, *Diplodinium*, and *Ophryoscolex* were classified as LCR. In the estimated absolute abundance dataset, 14 bacterial genera (RFT2d: 31.28% and RFT7d: 28.95%) and 5 bacterial genera (RFT2d: 1.19% and RFT7d: 0.93%) were categorized into CCR and InCCR taxa, respectively; 24 bacterial genera (RFT2d: 7.95% and RFT7d: 8.20%) were classified as GCR taxa and 22 bacterial genera (RFT2d: 13.96% and RFT7d: 13.77%) were classified as LCR taxa (Fig. 3B2: RFT2d and B4: RFT7d). Similarly, no archaeal and protozoal taxa were categorized as CCR, InCCR, GCR, and LCR taxa, except for 1 archaeal species (RFT2d: 53.16%; Fig. 3B6 and RFT7d: 43.78%; Fig. 3B8) that was classified as GCR. The circadian parameters of the abovementioned taxa were included in the Suppl File S2 (sheet 4).

### The circadian rhythm of predicted functions of bacteria and archaea

**Fig. 4:**
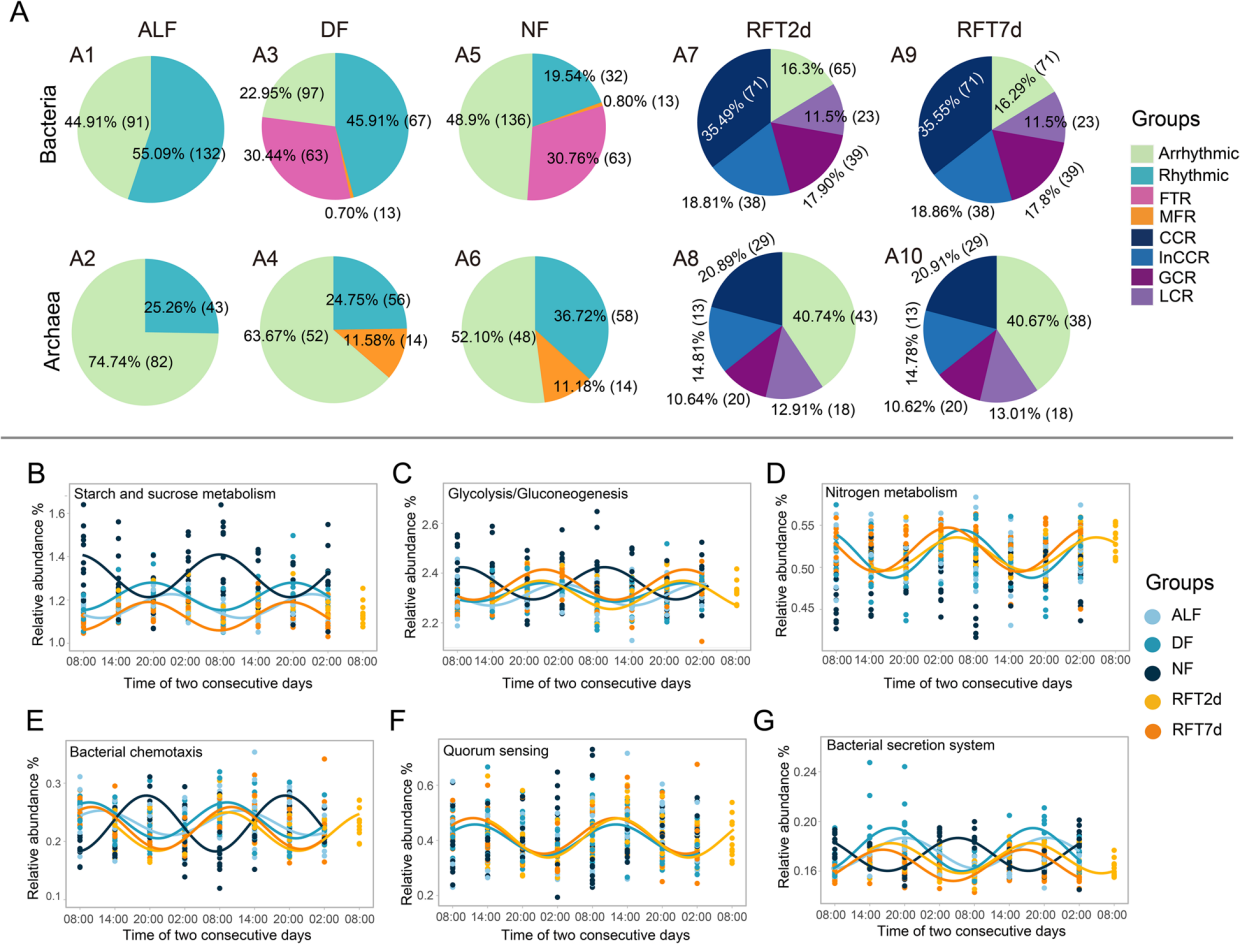
The circadian rhythm of bacterial and archaeal function. **A** The proportion of different circadian rhythm patterns of microbial pathways. The daily pattern of the relative abundance of starch and sucrose metabolism (**B**), clycolysis/gluconeogenesis (**C**), nitrogen metabolism (**D**), bacterial chemotaxis (**E**), quorum sensing (**F**), and bacterial secretion system (**G**). ALF, ad libitum feeding; DF, daytime feeding; NF, nighttime feeding; RFT2d, 48 h after rumen fluid transplantation. RFT7d, 7 days after rumen fluid transplantation. FTR, feeding time responsive. MFR, multi-factor responsive taxa. CCR, consistent circadian rhythm. InCCR, inconsistent circadian rhythm taxa. GCR, gain circadian rhythm. LCR, loss of circadian rhythm taxa

Circadian patterns were also identified for the bacterial and archaeal communities’ predicted functions using phylogenetic investigation of communities by reconstruction of unobserved states 2.0 (PICRUSt 2.0) [35]. Similar to the circadian rhythm of the relative abundance of microbial taxa, the circadian rhythm of the relative abundance of microbes’ functional pathways also showed different patterns under distinct feeding regimes and after rumen fluid transplantation conditions. Under the ALF regime, the relative abundances of 132 (55.09%; Fig. 4A1) and 43 (25.26%; Fig. 4A2) pathways (level 3 functions) in bacterial and archaeal communities, respectively, were rhythmic. Under feeding-restricted regimes, 63 (DF: 30.44%; Fig. 4A3 and NF: 30.76%; Fig. 4A5) bacterial pathways were classified as FTR, while 13 (DF: 0.70% and NF: 0.80%) bacterial and 14 archaeal (DF: 11.58%; Fig. 4A4 and NF: 11.18%; Fig. 4A6) pathways were classified as MFR. Under rumen fluid transplantation condition, 71 (RFT2d: 35.49% and RFT7d: 35.55%), 38 (RFT2d: 18.81% and RFT7d: 18.86%), 39 (RFT2d: 17.90% and RFT7d: 17.80%), and 23 (RFT2d: 11.50% and RFT7d: 11.50%) pathways in bacterial community were classified as consistent, inconsistent, gain, and loss circadian rhythm from RFT2d (Fig. 4A7) to RFT7d (Fig. 4A9) groups, respectively. In archaeal community, 29 (RFT2d: 20.89% and RFT7d: 20.91%), 13 (RFT2d: 14.81% and RFT7d: 14.78%), 20 (RFT2d: 10.64% and RFT7d: 10.62%), and 18 (RFT2d: 12.91% and RFT7d: 13.01%) pathways were categorized as consistent, inconsistent, gain, and loss circadian rhythm from RFT2d (Fig. 4A8) to RFT7d (Fig. 4A10) groups, respectively. The circadian parameters of the abovementioned pathways were presented in the supplement dataset (Suppl File S3).

Among the abovementioned pathways, the circadian rhythms of the relative abundance of starch and sucrose metabolism (Fig. 4B), glycolysis/gluconeogenesis (Fig. 4C), bacterial chemotaxis (Fig. 4E), and the bacterial secretion system (Fig. 4 G) pathways were classified as FTR under feeding restriction regimes. In addition, the circadian rhythms of nitrogen metabolism (Fig. 4D), bacterial chemotaxis (Fig. 4E), and quorum sensing (Fig. 4F) pathways were classified as CCR under rumen fluid transplantation conditions. The circadian rhythms of the glycolysis/gluconeogenesis and bacterial secretion system pathways were classified as InCCR. Among these rhythmic pathways, the relative abundances of starch and sucrose metabolism, glycolysis/gluconeogenesis, and bacterial secretion system pathways were increased after feeding and their peak time in the rhythm was at about after feeding 12 h. The relative abundances of nitrogen metabolism and bacterial chemotaxis pathways were decreased after feeding, and their peak time in the rhythm was at about after feeding 24 h. The peak time in the rhythm of quorum sensing pathways was at about 4 h after feeding in the DF, RFT2d, and RFT7d groups.

**Fig. 5:**
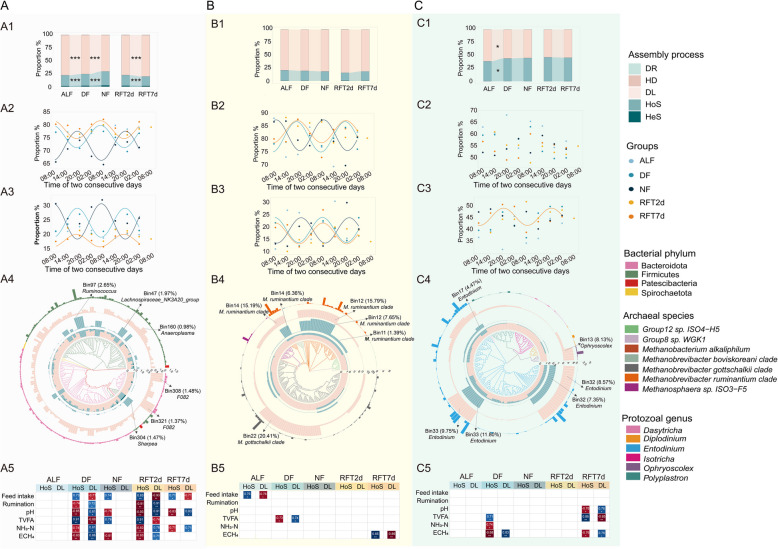
The daily patterns of bacterial (**A**), archaeal (**B**), and protozoal (**C**) community assembly process. The bacterial (A1), archaeal (B1), and protozoal (C1) community assembly process in ALF, DF, NF, RFT2d, and RFT7d groups. The significant differences between different groups were calculated by permutational t-test (1000 times). * *P* < 0.05, ** *P* < 0.01, and *** *P* < 0.001. The daily patterns of the proportion of dispersal limitation assembly process in bacterial (A2), archaeal (B2), and protozoal (C2) communities. The daily patterns of the proportion of homogeneous selection assembly process in bacterial (A3), archaeal (B3), and protozoal (C3) communities. Variation of ecological process across different phylogenetic groups in bacterial (A4), archaeal (B4), and protozoal (C4) communities. Phylogenetic tree was displayed at the center, and only relative abundance > 0.1% ASVs was presented in bacterial (A4), as well as > 0.01% ASVs was presented in archaeal (B4), and protozoal (C4) communities. From the inner to the outside, the 1st annulus presented the relative abundance of different ecological processes in each bin. The 2nd and 3rd annulus indicated the contribution of each bin to the whole community’s homogeneous selection and dispersal limitation processes, respectively. The 4th annulus represents the average relative abundance of each bin. The correlation between the (A5) bacterial, (B5) archaeal, and (C5) protozoal assembly process and feed intake, as well as rumination and rumen fermentation parameters. Only the significant correlations were presented in the figures. The deterministic processes included heterogeneous selection (HeS) and homogeneous selection (HoS). The stochastic process included homogenizing dispersal (HD), dispersal limitation (DL), and drift and others (DR). ALF, ad libitum feeding; DF, daytime feeding; NF, nighttime feeding; RFT2d, 48 h after rumen fluid transplantation; RFT7d, 7 days after RFT

### Rumen microbial assembly processes also showed circadian rhythms

Both stochastic and deterministic processes were found in the rumen bacterial, archaeal, and protozoal communities, which also showed rhythmic dynamics under different feeding regimes and after rumen fluid transplantation. Within the stochastic process, the proportion of dispersal limitation (DL) process ranged from 70 to 80% (ALF: 77.79%, DF: 75.35%, NF: 70.20%, RFT2d: 77.42% and RFT7d 79.94%), 79 to 85% (ALF: 79.44%, DF: 80.63%, NF: 81.76%, RFT2d: 84.16% and RFT7d 81.67%), and 53 to 62% (ALF: 61.35%, DF: 55.91%, NF: 54.99%, RFT2d: 53.65% and RFT7d 54.43%), for bacterial, archaeal, and protozoal communities, respectively (Fig. 5A1, B1, and C1) and no homogenizing dispersal (HD) and drift and others (DR) processes was found. Within the deterministic process, the proportion of homogeneous selection (HoS) process was in the range 18 to 27% (ALF: 20.73%, DF: 22.76%, NF: 26.98%, RFT2d: 20.51% and RFT7d 18.27%), 15 to 21% (ALF: 20.17%, DF: 19.08%, NF: 18.00%, RFT2d: 15.22% and RFT7d 18.01%), and 38 to 47% (ALF: 38.52%, DF: 44.04%, NF: 44.99%, RFT2d: 46.32% and RFT7d 45.44%), and the proportion of heterogeneous selection (HeS) was in the range 1 to 2%, < 1%, and < 1% in bacterial, archaeal, and protozoal communities, respectively (Fig. 5A1, B1, and C1).

The proportion of the DL process was lower (*P* < 0.001), while the HoS process was higher (*P* < 0.001) in the DF group compared to the ALF group, and in the NF group compared to the DF group, as well as in the RFT7d group than the RFT2d group (Fig. 5A1). No difference in the proportion of DL and HoS processes was observed in the archaeal community among these comparisons (Fig. 5B1). The proportion of the DL process was lower (*P* < 0.001), while the HoS process was higher (*P* < 0.05) in the DF group compared to the ALF group in the protozoal community (Fig. 5C1). Furthermore, the proportions of DL and HoS processes in bacterial and archaeal communities were rhythmic in the DF, NF, and RFT7d groups. For the bacterial community, the peak time in the rhythm of the DL process was at 08:00, 20:00, and 08:00, while the peak time in the rhythm of the HoS process was at 20:00, 08:00, and 20:00 in the DF, NF, and RFT7d groups (Fig. 5A2 and A3). Daily patterns of assembly process in the archaeal community were on the contrary (the peak time-shifted 12 h) with a bacterial community (Fig. 5B2 and B3). Moreover, the proportion of the HoS process was rhythmic in the RFT7d group in the protozoal community, and its peak time was at 20:00 (Fig. 5C3).

### Identification of microbes contributing to microbial community assembly processes

**Fig. 6:**
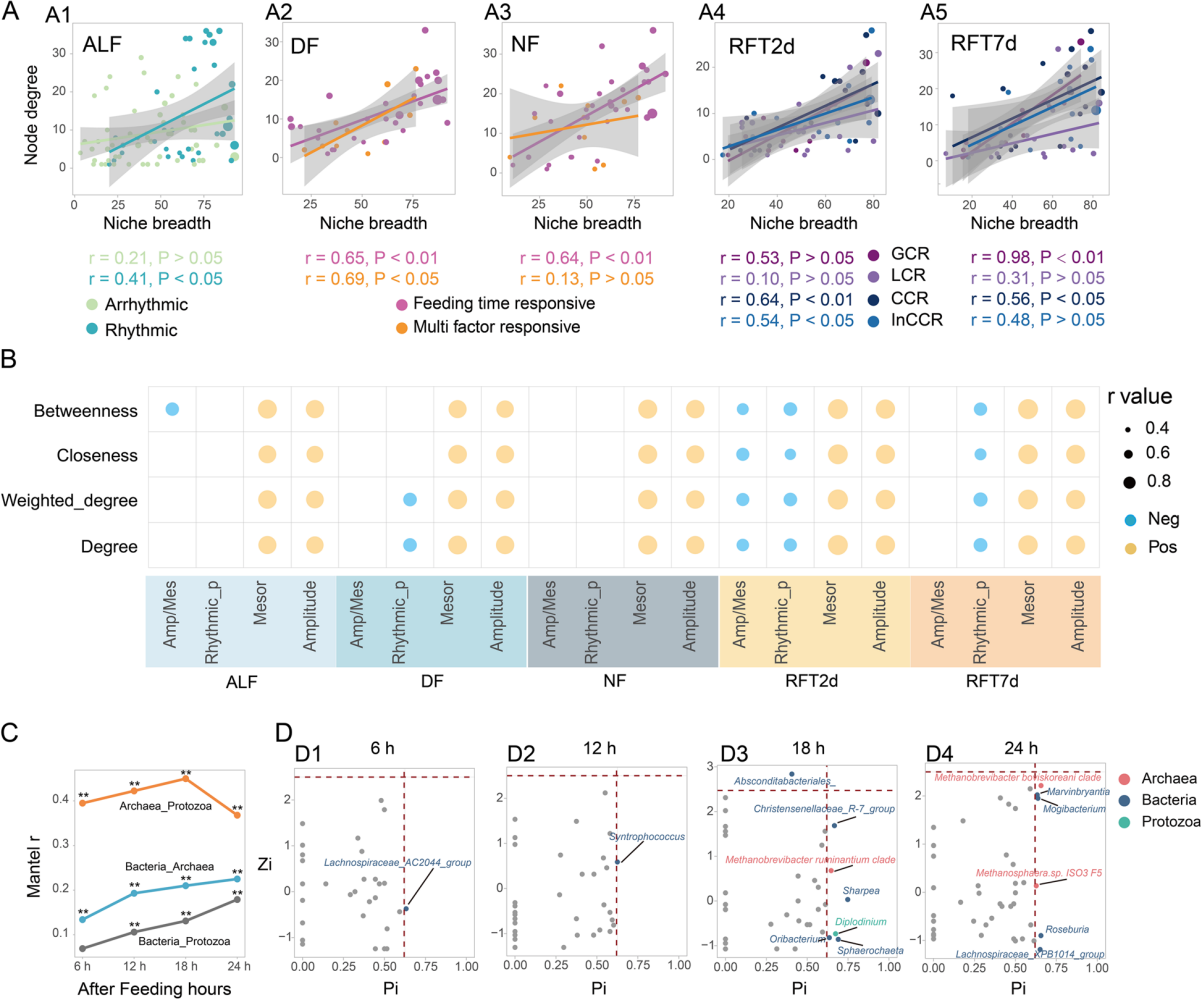
Ruminal microbial networks node features and their correlation with niche breadth and circadian rhythm parameters. A Spearman correlations between ruminal microbiota network node degree and microbial taxa’s niche breadth. B Spearman correlations between ruminal microbiota network node feature and circadian parameters. Only r > 0.40 and *P* < 0.05 correlations were shown in the figure. Amp/Mes, amplitude/mesor; Rhythmic_p, the *P* value of Circacompare. C The relationship among bacteria, archaea, and protozoa based on the Mantel test, * *P* < 0.05, ** *P* < 0.01. D Distribution of ruminal bacteria genera, protozoa genera, and archaea species based on their network roles, generalists including network hubs (Zi > 2.5, Pi > 0.62), module hubs (Zi > 2.5, Pi < 0.62), and connectors (Zi < 2.5, Pi > 0.62); specialists: peripherals (Zi < 2.5, Pi < 0.62). Zi: within-module connectivity, Pi: among-module connectivity

We identified microbes that contributed to HoS and DL processes based on a binning approach [39] (See Methods) (Suppl File S4). In the bacterial community, the top 3 bins that contributed to the DL process were bin308 (genus *F*082, 1.48%), bin321 (genus *F*082, 1.37%), and bin160 (genus *Anaeroplasma*, 0.98%) (Fig. 5A4). The top 3 bins that contributed to the HoS process were bin97 (genus *Ruminococcus*, 2.65%), bin47 (genus *Lachnospiraceae_NK*3*A*20*_group*, 1.97%), and bin304 (genus *Sharpea*, 1.47%), respectively (Fig. 5A4). In archaeal community, the top 3 bins contributed to DL process were bin22 (species *Methanobrevibacter gottschalkii clade*, 20.41%), bin12 (species *Methanobrevibacter ruminantium clade*, 15.79%), and bin14 (species *Methanobrevibacter ruminantium clade*, 15.19%), while the top 3 bins contributed to the HoS process were bin12 (species *Methanobrevibacter ruminantium clade*, 7.65%), bin14 (species *Methanobrevibacter ruminantium clade*, 6.36%), and bin11 (species *Methanobrevibacter ruminantium clade*, 1.39%) (Fig. 5B4). In the protozoal community, the top 3 bins contributed to the DL process were bin33 (genus *Entodinium*, 9.75%), bin32 (genus *Entodinium*, 8.57%), and bin13 (genus *Ophryoscolex*, 8.13%), while the top 3 bins contributed to the HoS process were bin33 (genus *Entodinium*, 11.80%), bin32 (genus *Entodinium*, 7.35%), and bin17 (genus *Entodinium*, 4.47%) (Fig. 6C4). Among these microbial taxa, the bacterial genus *F*082, *Ruminococcus*, *Sharpea*, and protozoal genus *Entodinium* were classified as FTR taxa, while the bacterial genus *Anaeroplasma* was classified as MFR taxa.

#### The associations between microbial assembly process and feed intake, rumination time, and ruminal fermentation parameters

Among the observed significant correlations, we found that the proportion of HoS process was positively correlated (*r* > 0.7, *P* < 0.05) with feed intake and total volatile fatty acid (TVFA) concentration, and negatively associated (*r* < − 0.7, *P* < 0.05) with rumination time, ruminal pH, NH_3_-N concentration, and estimated methane (calculated based on molar amount of methane molecular generated when one molar of TVFA was produced, ECH_4_) [47] in bacterial community (Fig. 5A5). The proportion of the HoS process was positively correlated (*r* ≥ 0.76, *P* < 0.05) with feed intake and ECH_4_, and negatively associated (*r* = − 0.73, *P* < 0.05) with TVFA concentration in the archaeal community (Fig. 5B5). Similar to bacteria, the proportion of the HoS process was positively associated (*r* ≥ 0.71, *P* < 0.05) with TVFA concentration, negatively associated (*r* ≤ − 0.76, *P* < 0.05) with ruminal pH, NH_3_-N, and ECH_4_ in protozoal communities (Fig. 5C5). The proportion of the DL process had the opposite correlation compared to the HoS process with feed intake, rumination time, and rumen fermentation parameters in bacterial, archaeal, and protozoal communities.

### Relationship between microbial taxa node features and niche breadth, as well as their circadian rhythm parameters

We found that microbial interactions can be changed at different times of the day. Furthermore, the node features of the taxa in the network were associated with their niche breadth, and these associations can be different for the taxa with different circadian rhythm patterns. Microbial community networks revealed the number of edges and average degrees were 479, 349, 563, 349, and 457; 4.54, 3.31, 5.34, 3.31, and 4.33, in ALF, DF, NF, RFT2d, and RFT7d groups, respectively. Further analysis of the association between microbial taxa node degree in the network and their niche breadth showed a positive correlation (*r* = 0.41, *P* < 0.05) between the node degree and their niche breadth of rhythmic taxa in the ALF group (Fig. 6A1). However, this correlation was not significant in arrhythmic taxa. Niche breadth was positively correlated with the node degree of FTR taxa in DF (*r* = 0.65, *P* < 0.01) and NF (*r* = 0.64, *P* < 0.01) groups (Fig. 6A2 and A3). Only a positive correlation was observed between niche breadth and node degree of MFR taxa in the DF (*r* = 0.69, *P* < 0.05) group (Fig. 6A2). The CCR taxa showed a positive correlation between their niche breadth and node degree in both RFT2d (*r* = 0.64, *P* < 0.01) and RFT7d (*r* = 0.56, *P* < 0.05) groups; however, the InCCR taxa only had such significant correlation in the RFT2d (*r* = 0.54, *P* < 0.05) group (Fig. 6A4 and A5). A significantly positive correlation (*r* = 0.98, *P* < 0.01) was observed between niche breadth and node degree of GCR taxa in the RFT7d group (Fig. 6A5); nevertheless, this relationship was not found in the RFT2d group.

Furthermore, we found that the network’s node features were associated with microbial taxa’s circadian patterns. The mesor and amplitude of the microbial circadian rhythm showed a significantly positive correlation (*r* > 0.60, *P* < 0.01) with network node features (degree, weighted degree, closeness, and betweenness) in ALF, DF, NF, RFT2d, and RFT7d groups (Fig. 6B). The amplitude-to-mesor ratio had a significantly negative correlation with the node betweenness in ALF (*r* = 0.43, *P* < 0.01) and RFT2d (*r* = − 0.52, *P* < 0.01) groups. A smaller *P*-value in the cosine model indicates a more pronounced statistical significance of the rhythm. The microbes’ rhythmic *P* values were negatively correlated with the node degree, weighted degree, closeness, and betweenness (*r* < − 0.47, *P* < 0.01) in RFT2d and RFT7d groups. In the DF group, the rhythmic *P* value also significantly correlated with the node degree and node weighted degree (*r* < − 0.55, *P* < 0.01).

### Dynamic relationships among the rumen microbes

At the community level, we found that the correlations (Mantel *r*-value) between archaeal and protozoal communities were stronger compared to the correlation between bacterial and archaeal/protozoal communities (Fig. 6C). Additionally, the archaeal community had a stronger correlation with the bacterial community than the protozoal community. The correlations between bacterial and archaeal/protozoal communities were increased after feeding hours prolongation from 6 to 24 h. The correlation between archaeal and protozoal communities increased after feeding from 6 to 18 h; however, it decreased from 18 to 24 h. Further analysis of microbial networks showed a number of edges and average degree were 391, 439, 357, and 486; 4.23, 5.08, 4.15, and 5.40, in the networks of 6, 12, 18, and 24 h after feeding groups, respectively. Additionally, we found that the network generalists (the nodes that were highly correlated with the other nodes within the same module and/or out of the module) increased with the after-feeding hours prolonged (Fig. 6D). Specifically, only one generalist was observed after feeding 6 h (Fig. 6D1) and 12 h (Fig. 6D2), respectively, while there were 7 and 6 generalists in the networks after feeding 18 h (Fig. 6D3) and 24 h (Fig. 6D4) groups.

### The circadian rhythm of feed intake, rumination, and rumen fermentation parameters

**Fig. 7:**
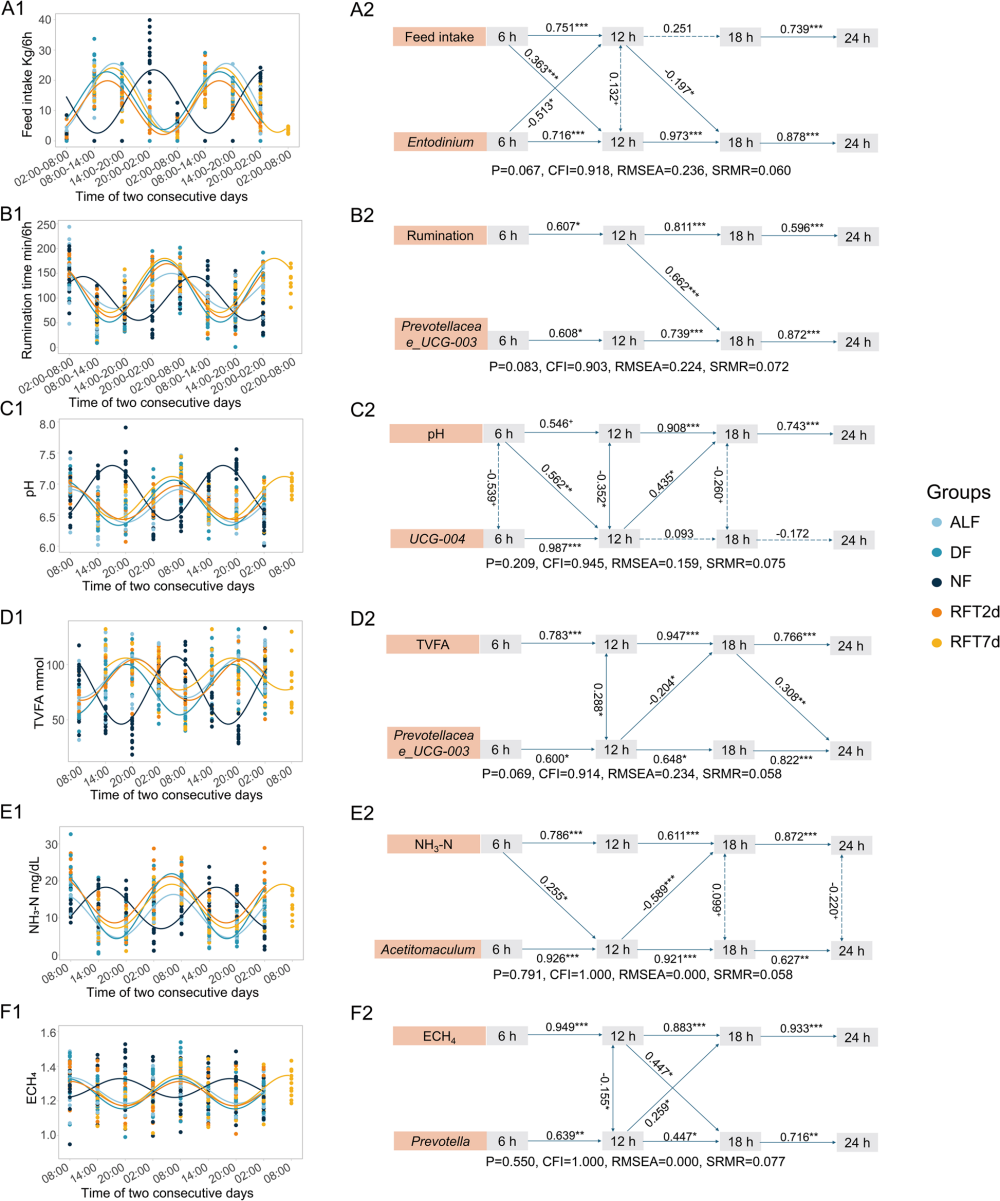
The daily patterns of feed intake, rumination time, and ruminal fermentation parameters and their cross-lagged correlation with microbial taxa. The daily patterns of feed intake (A1), rumination time (B1), ruminal pH (C1), TVFA concentration (D1), NH3-N concentration (E1), and ECH4 (F1) were presented with the cosinor fitted curve. The cross-lagged panel model analysis revealed the relationships between the relative abundance of microbial taxa and feed intake (A2), rumination time (B2), ruminal pH (C2), TVFA concentration (D2), NH3-N concentration (E2), and ECH4 (F2). Only the significant path coefficient and informative correlations were kept in the figures. The solid line represents a significant correlation, while the dashed line represents a trend significance. 0.05 < + *P* < 0.01, * *P* < 0.05, ** *P* < 0.01, and *** *P* < 0.001. The 6 h, 12 h, 18 h, and 24 h represent the after-feeding hours. ALF, ad libitum feeding; DF, daytime feeding; NF, nighttime feeding; RFT2d, 48 h after rumen fluid transplantation; RFT7d, 7 days after RFT. TVFA, total volatile fatty acids; NH3-N, ammonia nitrogen; ECH4, estimated methane; CFI, comparative fit index; RMSEA, root-mean-square error of approximation; SRMR, standardized root-mean-square residual

The daily patterns of feed intake (Fig. 7A1), rumination time (Fig. 7B1), ruminal pH (Fig. 7C1), acetate, propionate, butyrate, valerate, TVFA (Fig. 7D1), acetate/propionate (A/P) ratio, NH_3_-N (Fig. 7E1), and ECH_4_ (Fig. 7F1) were rhythmic in ALF, DF, NF, RFT2d, and RFT7d groups (Suppl File S5 and Fig. 7). The peak time in the rhythm of feed intake, acetate, propionate, butyrate, valerate, and TVFA concentration was in the evening (16:00–20:00), while the peak time in the rhythm of rumination, pH, A/P ratio, NH_3_-N, and ECH_4_ was in the morning (05:00–09:00) in ALF, DF, RFT2d, and RFT7d groups. The peak time in the rhythm of the aforementioned parameters was shifted from morning to evening and/or from evening to morning in the NF group compared to the DF group. However, the peak time in the rhythm of rumination was at 10:42 in the NF group, which was a 5 h shift compared to at 05:18 in the DF group. In addition, the ruminal isobutyrate concentration was only rhythmic in the DF (peak time 19:17), NF (05:07), and RFT7d (03:40) groups, while the isovalerate concentration was rhythmic in the NF (14:22) and RFT7d (01:32) groups.

### The cross-lagged relationships between feed intake, rumination time, rumen fermentation, and rumen microbiota

The CLPM analysis showed a good fit of the cross-lagged relationships between the relative abundance of rumen microbial taxa and feed intake, as well as rumination time and rumen fermentation parameters (Suppl File S6). In this model, three types of correlations were assessed: (1) auto-regressive correlation: the correlation between a variable measured at two consecutive time points, (2) co-occurring association: the correlation between two variables measured at the same time point, and (3) cross-lagged correlation: the correlation between two variables measured at different time points, where the earlier value of each variable is thought to affect the later value of the other variable [48]. The autoregressive correlation (*β* > 0.46, *P* < 0.001) indicated high temporal stability for rumination time, ruminal fermentation parameter, and the relative abundance of the protozoal genus *Entodinium*, bacterial genera *Prevotella*, *NK*4*A*214*_group*, *Prevotellaceae_UCG-*001, and *Mogibacterium*, *g_unclassified_f_Lachnospiraceae*, *Acetitomaculum*, *Prevotellaceae_UCG-*003, and *Clostridia_UCG-*014 (Suppl File S6). No significant co-occurring association was observed between the feed intake, rumination, and the relative abundance of microbial taxa. However, a co-occurring association was observed between the relative abundance of microbial taxa and fermentation parameters, such as the relative abundance of *UCG-*004 was negatively associated (*β* = − 0.35, *P* < 0.05) with pH after feeding for 12 h (Fig. 7C2).

The cross-lagged correlation revealed significant cross-lagged influences between feed intake, rumination time, rumen fermentation, and rumen microbiota. Specifically, the feed intake after feeding 0 and 6 h had a lagged positive correlation (*β* = 0.36, *P* < 0.01) with the relative abundance of *Edtodinium* after feeding 12 h (Fig. 7A1). On the other hand, the relative abundance of *Edtodinium* after feeding 6 h had a lagged negative correlation (*β* = − 0.53, *P* < 0.05) with feed intake between after feeding 6 and 12 h. The rumination time after feeding 6 and 12 h had a lagged positive association (*β* = 0.66, *P* < 0.05) with the relative abundance of *Prevotellaceae_UCG-*003 after feeding 18 h (Fig. 7B2). Ruminal pH after feeding 6 h had a lagged positive correlation (*β* = 0.56, *P* < 0.01) with the relative abundance of *UCG-*004 after feeding 12 h and the relative abundance of *UCG-*004 at after feeding 12 h had a lagged positive correlation (*β* = 0.44, *P* < 0.05) with pH after feeding 18 h (Fig. 7C2). The TVFA concentration after feeding 18 h had a lagged positive correlation (*β* = 0.31, *P* < 0.01) with the relative abundance of *Prevotellaceae_UCG-*003 after feeding 24 h (Fig. 7D2). The relative abundance of *Acetitomaculum* after feeding 12 h had a lagged negative correlation (*β* = − 0.60, *P* < 0.001) with NH_3_-N concentration after feeding 18 h (Fig. 7E2). The ECH_4_ after feeding 12 h had a lagged positive correlation (*β* = 0.45, *P* < 0.05) with the relative abundance of *Prevotella* after feeding 18 h (Fig. 7F2).

### The inferred causal relationships among feed intake, rumination time, fermentation parameters, rumen microbiota diversity, and population

**Fig. 8:**
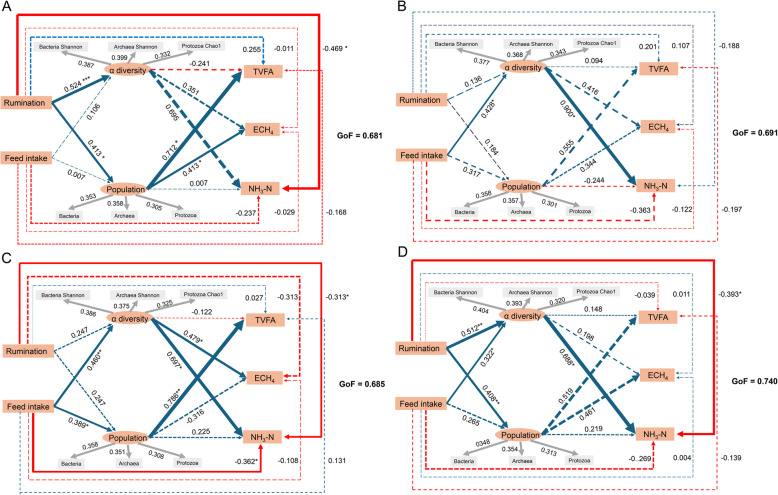
The cause-and-effect relationship among feed intake, as well as ruminations, microbiota, and ruminal fermentation parameters at different after-feeding hours. The data after feeding for 6 h (A), 12 h (B), 18 h (C), and 24 h (D) were analyzed, respectively. Observed variables are represented in a rectangular form, the latent variables are represented in an elliptical form. Redline indicated a negative correlation, and the blueline indicated a positive correlation. The line thickness indicated the coefficient value, and the significance of the coefficient was tested by bootstrap, * *P* < 0.05, ** *P* < 0.01. TVFA: total volatile fatty acids, NH3-N: ammonia nitrogen, ECH4: estimated methane. GoF: goodness of fitness. PLS-PM: partial least squares path modeling

The Shannon index of bacteria and archaea had a higher absolute regression coefficient with feed intake, rumination, microbial population, and ruminal fermentation parameters than the Chao1 index, while the protozoal Chao1 had a higher absolute regression coefficient with these indices than Shannon (Fig. S6 A). Therefore, we chose bacterial Shannon, archaeal Shannon, and protozoal Chao1 as the representative variables for ruminal microbial alpha diversity to conduct the inferred causal relationship analysis with PLS-PM. The goodness of fit in the PLS-PM was 0.68, 0.69, 0.69, and 0.74 in the after-feeding 6 (Fig. 8A), 12 (Fig. 8B), 18 (Fig. 8C), and 24 (Fig. 8D) h groups, respectively (Fig. 8).

Rumination time was positively associated with rumen microbial diversity after feeding 6 h (*β* = 0.52, *P* < 0.001) and 24 h (*β* = 0.51, *P* < 0.001), respectively. Additionally, the feed intake was positively associated with microbial diversity after feeding 12 h (*β* = 0.43, *P* < 0.05), 18 h (*β* = 0.46, *P* < 0.01), and 24 h (*β* = 0.32, *P* < 0.05), respectively. Rumination time was also positively correlated with rumen microbial population after feeding 6 h (*β* = 0.41, *P* < 0.05) and 24 h (*β* = 0.41, *P* < 0.01), respectively, while the feed intake was positively associated with microbial population after feeding 18 h (*β* = 0.46, *P* < 0.01). The TVFA concentration was positively related to the microbial population after feeding for 6 h (*β* = 0.71, *P* < 0.05) and 18 h (*β* = 0.79, *P* < 0.01), respectively, while no significant correlation was observed between microbial diversity and TVFA concentration. The ECH_4_ was positively associated with microbial population (*β* = 0.71, *P* < 0.01) and microbial diversity (*β* = 0.48, *P* < 0.01) after feeding for 6 h and 18 h, respectively. The NH_3_-N concentration was positively correlated with microbial diversity after feeding for 12 h (*β* = 0.90, *P* < 0.05), 18 h (*β* = 0.70, *P* < 0.05), and 24 h (*β* = 0.69, *P* < 0.05), respectively and no significant correlation was observed between microbial population and NH_3_-N concentration. Moreover, we found that the NH_3_-N concentration was negatively associated with rumination time after feeding for 6 h (*β* = − 0.47, *P* < 0.05), 18 h (*β* = − 0.31, *P* < 0.05), and 24 h (*β* = − 0.39, *P* < 0.05), respectively, as well as feed intake after feeding for 18 h (*β* = − 0.36, *P* < 0.05).

The inferred causal relationships among feed intake, rumination time, rumen fermentation, microbial diversity, and population were further assessed for bacterial (Fig. S6 B), archaeal (Fig. S6 C), and protozoal (Fig. S6 D) communities, respectively. The feed intake had a positive association with archaeal diversity (*β* = 0.49, *P* < 0.01), while the rumination time had a positive association with protozoal diversity (*β* = 0.42, *P* < 0.01). Furthermore, the population of prokaryotes showed a positive correlation with rumen TVFA concentration (bacteria: *β* = 0.85, *P* < 0.001 and archaea: *β* = 0.74, *P* < 0.001) and ECH_4_ (bacteria: *β* = 0.71, *P* < 0.001 and archaea: *β* = 0.61, *P* < 0.001), while the population of eukaryote (protozoa) was not. However, the alpha diversity of protozoa was positively correlated with ECH_4_ (*β* = 0.42, *P* < 0.01). Finally, the archaeal population also showed a positive association with NH_3_-N concentration (*β* = 0.40, *P* < 0.05).

## Discussion

In this study, we identified the circadian rhythm of rumen microbes’ population, abundance, and diversity under different feeding regimes as well as after the introduction of exogenous microbiota by the rumen fluid transplantation. Such study design is novel and has allowed the assessment of the “true” circadian rhythm of rumen microbes and how they can be affected by external factors such as feeding time. Similar to the previous studies reported circadian rhythms of microbes in the gut of mammals (humans, pigs, and dairy cows) [15, 16, 51], we found the circadian rhythm of bacteria and archaea in the rumen, confirming the evidence of the diurnal oscillation of gut prokaryotes. To date, significant knowledge has been established on the effects of feeding patterns and diet composition on rumen protozoa [52]. Our findings on the circadian rhythm of protozoal populations and composition under time-restricted feeding and the introduction of exogenous microbes suggested that in addition to their chemotaxis, the internal factors could also be one of the driving factors influencing protozoa’s dynamics in vivo.

Under the feeding restriction trials (DF vs. NF), we further classified the rhythmic microbial taxa as FTR and MFR taxa. In line with previous studies in the fecal bacteria of mice [16], the circadian rhythm of the majority of ruminal bacteria can be FTR. The daily feeding patterns can change the nutrient concentration in ruminal digesta suggesting the changes in circadian patterns of FTR taxa may be more related to their sensitivity to the nutrient and substrate dynamics in the rumen through the feeding cycle. The change in circadian patterns of MFR taxa did not align with the shifting feeding time, suggesting that multiple endogenous and exogenous factors could influence the dynamic shift of these bacterial taxa. For example, the bacterial genus *Anaeroplasma*, which has been reported to be positively correlated with ruminal acetate concentrations [53], was classified as MFR. Previous study reported that rumen *Anaeroplasma* is heritable in beef cattle [13]. Therefore, the host genome may directly affect the relative abundance of this taxon, which further led to inconsistent circadian pattern shifting of the relative abundance of *Anaeroplasma* with feeding time shifting as the cows were still kept in the same day-night (light–dark) cycle in the NF group compared to the DF group. We also found a significant correlation between the node degree and niche breadth in MFR taxa that disappeared under abnormal (night) feeding time (NF group) compared to normal (day) feeding time (DF group). Previous study reported that mistimed feeding (such as feeding at night) can disturb the circadian rhythm of clock gene expression (quantified in serum) and alter the bacterial composition in feces of growing pigs [54]. We speculate that the disturbed expression of host clock genes may affect cows’ physiology and metabolism [55, 56], as the results influence the MFR taxa’s niche breadth and/or MFR taxa-related microbial interactions, which warrants future studies.

The introduction of exogenous microbes can also alter the circadian rhythm of rumen microbiota. However, the discrepancy in their stability in response to the introduction of exogenous microbiota led to different circadian rhythm shifts (consistent, inconsistent, loss, and gain circadian rhythm). Previous studies reported the microbial taxa’s resistance (maintaining the same abundance) and resilience (return to pre-invasion abundance over time) to exogenous microbiota invasion in the human gut and environments [57, 58]. Our study clarified the resistance (consistent circadian rhythm) and resilience (adaptation of circadian rhythm) of taxa in the rumen, highlighting the need to consider the stability of endogenous taxa when introducing exogenous microbiota to the rumen. The observed consistent circadian rhythm for bacteria but not for archaea and protozoa after rumen fluid transplantation suggests the higher stability of the bacterial community. The niche breadth [59] and inter-microbial interactions [60] within a microbial community are the key factors that affect the microbial taxa’s stability. Previous study reported that the generalists (ones highly interacting with other taxa) normally have wide habitat preferences [61]. Our study further confirmed that the taxon’s niche breadth was positively correlated with network node degree, confirming this observation. However, in our rumen fluid transplantation study, only these consistent circadian bacterial taxa showed that their niche breadth and node degree had significantly positive correlations in both RFT2d and RFT7d groups. We speculated that these taxa may have a broader niche breadth and more stable interactions with other taxa, that is why they can keep consistent circadian patterns after introducing exogenous microbiota. Further studies are required to further validate these speculations.

In the archaeal community, we found methylotrophic methanogens were feeding time responsive, such as species Group 8 sp. WGK1 and *Methanosphaera *sp. ISO3-F5. This may be attributed that feedstuffs contain methyl [62], which can directly supply substrates for methylotrophic methanogens [62–64]. Furthermore, it has been reported that the diet supplemented with seaweed can decrease the expression of methyl coenzyme-M reductase subunit genes of methylotrophic methanogens [65]. The observed peak time (19:00 under ad libitum conditions) of the relative abundance of *Methanosphaera *sp. ISO3-F5 (with a relative abundance > 6% of total methanogens, Suppl File S2) may provide insights into the optimal feeding time or the digestibility rate of feedstuffs associated with these methylotrophic methanogens, which warrant future studies. However, acetoclastic and hydrogenotrophic methanogens, such as *Methanobrevibacter gottschalkii clade* and *Methanobrevibacter ruminantium clade*, were not classified as FTR taxa. The acetate and hydrogen were produced by bacterial taxa, which were further utilized by the acetoclastic and hydrogenotrophic methanogens to produce methane [11], suggesting the daily dynamics of acetoclastic and hydrogenotrophic methanogens are more dependent on the bacteria’s metabolism and indirectly affected by substrates in the rumen. In addition, the circadian rhythm of MFR taxon Group 12 sp. ISO4-H5 showed good resilience (gain circadian rhythm from the RFT2d to RFT7d groups) after rumen fluid transplantation suggesting that it can adapt effectively to the introduction of exogenous microbes. Our results indicate that shifting the feeding time could be an alternative approach to affect methane emission by manipulating the methylotrophic methanogens and targeting other microbial taxa could be a strategy to indirectly affect the acetoclastic and hydrogenotrophic methanogens.

Compared to bacteria and archaea, protozoa had the largest proportion of FTR taxa, suggesting that they were more sensitive to nutrient and substrate dynamic shifts through the digestion and feeding cycle. Protozoa have been reported to have chemotaxis and migratory features in the rumen, and these functions were more active in the rumen when the cows were under fast conditions [66]. Our results showed that the protozoal genus *Entodinium* had a cross-lagged correlation with feed intake, particularly the time within 0 to 12 h after feeding, suggesting that FTR protozoa are more responsive to the substrates’ availability. The necessity of ruminal protozoa is debatable because defaunation research showed that removal of protozoa increased [67] or decreased [68] the cellulolytic bacteria, as well as affected overall rumen fermentation [69]. Due to the lack of detection of MFR protozoa, we speculated that rumen protozoa could be independent of cattle host daily dynamics but are highly dependent on the diet. It has been reported that protozoa contributed to the maintenance of bacterial diversity in the rumen [20], highlighting the importance of protozoa in affecting other rumen microbes and their functions. Additionally, our analysis revealed that the archaea had a higher correlation with protozoa than with bacteria, supporting the well-established understanding that methanogens form more active symbiotic relationships with protozoa compared to bacteria [70]. Previous studies indicated that the methanogenic taxa associated with rumen ciliates were responsible for between 9 and 25% methanogenesis in rumen fluid [71]. Our results indicated that the diversity (Shannon and Chao1) of protozoa was positively associated with ECH_4_, which further clarified the promotion role of protozoal diversity, not its population, on methane emission in dairy cows.

In addition to the relative abundance of microbial taxa, the relative abundances of rumen microbiota metabolic and signal pathways were also rhythmic, which could affect the daily dynamic shifts of rumen fermentation. The starch and sucrose metabolism and glycolysis/gluconeogenesis pathways were classified as FTR pathways and were increased after feeding. In the normal feeding cycle of lactating dairy cows, starch concentration increases after feeding [72] directly supplying substrate for microbial starch degradation and glycolysis in the rumen [10, 73]. The peak time in the rhythm of FTR taxa *Prevotella* and *Ruminococcus*, two genera reported as starch utilizers [74, 75], was close to the peak time of feed intake. This suggests that they may be the dominant taxa that contribute to the starch and sucrose metabolism and glycolysis/gluconeogenesis pathways, and the rhythmicity of these FTR taxa could be driven by the starch content in the rumen, which further promotes the TVFA production after feeding (Fig. 7D1). Similar to protozoa, our study further confirmed the chemotaxis of bacteria in responding to substrate change with bacterial chemotaxis function being classified as an FTR pathway and decreased after feeding. Previous study reported that colonization of ryegrass by fungi in the rumen was associated with chemotaxis [76]. Although rumen bacterial chemotaxis has been reported [77], our study added the document that bacterial chemotaxis had the peak time in the rhythm at 24 h after feeding (less nutritional substrate in the rumen), suggesting nutrient-driven bacterial activities. This result provided temporal dynamics information on the chemotactic ability of bacteria, which helps bridge the gap in future research on the colonization and degradation of feedstuffs by bacteria. Moreover, we predicted that quorum sensing (QS) was more active around 24 h after feeding with about 12 h shifting for the peak time in the rhythm of bacterial population and the relative abundance of quorum sensing (Fig. 4F). As QS is well known for intra- and inter-microbes communication and can be responsive to the increase of microbial population [78], indicating that in addition to population, the concentrations of nutrients in the rumen (changed with the time after feeding prolonging) could be also an important factor affecting the communications within the bacterial community. Increased Mantel *r* value between bacteria and archaea, bacteria and protozoa, and the number of generalists (taxa highly correlated with others within and across modules) when the after-feeding hours were prolonged, highlighting higher relative abundance of bacterial communication’s chemotaxis pathways may promote more microbial interactions when there was less nutrient substrate in the rumen (after feeding 24 h) (Fig. 6C and D).

Our study also highlighted the bidirectional interactions between the microbiota and rumen fermentation parameters, as well as feed intake and rumination. In line with previous study [79], our results revealed that feed intake and rumination time were positively associated with microbial diversity and population. Additionally, these associations can be dynamic within a day as the rumination time after feeding 0 to 6 h and the feed intake after feeding 12 to 18 h were significantly associated with both microbial diversity and population, respectively. After feeding for 6 h, ruminal pH has decreased and VFA concentration has increased. The rumination can help to make a favorable environment for rumen microbiota by removing carbon dioxide, and VFAs, and adding saliva to make the balance of pH [80]; therefore, the environmentally sensitive microbes can survive and improve the diversity of the rumen microbiota community. However, after feeding 18 h, the environmental conditions may be in balance as the pH started to increase, the microbes in rumen can survive and are more focused on the degradation and fermentation of substrates, that is why the feed intake (providing more substrates for microbiota) at after feeding 12 to 18 h can be significantly associated with rumen microbiota diversity and population. Our study suggests the daily dynamics of promotion roles of feed intake and rumination on the population and diversity of rumen microbiota, providing a more accurate understanding of the effects of the host feed intake and rumination on the rumen microbiota.

Moreover, lots of previous studies looked at the co-occurrence correlation between the microbial taxa and rumen fermentation parameters [81, 82]. However, the microbial taxa that had a significantly cross-lagged relationship with rumen fermentation suggest the carry-over effect of rumen microbes on VFA from the previous few hours. Similarly, the observed fermentation parameters were likely influenced by microbial taxa that were activated a few hours earlier. Traditionally, most rumen research has just linked the rumen microbiota with rumen fermentation using the rumen samples collected at the same time point. The identified lagged phase effect suggests that the relationship between rumen microbes and rumen fermentation is more complicated, and the sequential effect needs to be considered. Regardless, our findings have brought new insights into understanding the observed rumen fermentation and microbial taxa, which enhanced our ability to predict and manipulate rumen microbiota and fermentation in the future.

In addition to the daily dynamics of microbial composition and function, we found that assembly processes of rumen microbiota were rhythmic and associated with fermentation parameters. The observed HoS and DL processes indicated a combination of deterministic and stochastic assembly processes working synergistically for the ruminal microbiota assembly during the feeding cycle. Compared to bacteria (70–80%) and archaea (79–85%), the protozoa (53–62%) had the lowest percentage of the DL process. The DL process represents the restriction for microbial taxa to reach a new niche for colonization [83]. The lower DL processes of protozoa suggest that protozoa can be easier to move around and find their niche for colonization. Previous study reported that the DL process can constrain microbial taxa to better grow and utilize new nutritional substrates [83], while the HoS process can promote microbial taxa trade-off and/or collaboration [25]. Our study further confirmed that DL process was negatively associated with rumen fermentation, while the HoS process was positively correlated with the TVFA concentration in bacterial and protozoal communities (Fig. 5A5 and C5), suggesting that DL and HoS processes affected the colonization of rumen microbes in feedstuffs and altering microbial trade-off and/or collaboration, which in turn affect rumen fermentation. Additionally, the proportion of the DL process in the archaeal community was negatively associated with ECH_4_ in our study, suggesting that the DL process may limit the methanogens from producing methane. Among those top bins that contributed most to the assembly process, bacterial genus *F*082 (contributed to the DL process), *Ruminococcus* (HoS), *Sharpea* (HoS), and protozoal genus *Entodinium* (HoS and DL) were classified as FTR taxa, while the bacterial genus *Anaeroplasma* (DL) was classified as MFR taxa. These results provided a potential direction to harness these taxa to further shape the rumen microbial assembly process.

Lastly, our results also revealed that using relative abundance could reflect the quantitative dynamics of taxon-specific microbes as the difference in peak time was within 3 h between the relative abundance and estimated absolute abundance (Fig. S7). In line with the previous study complementing 16S rRNA gene amplicon sequencing with total bacterial load to infer absolute species concentrations could be a reasonable proxy of species-specific population values [84]. However, the estimated absolute abundance still has limitations in reflecting the bacteria at a low relative abundance [84] and missed the detection of the taxa they are dependent on the increase or decrease of other ones. Advanced technology, such as a synthetic DNA spike-in method for sequencing [85], is supposed to further measure the absolute abundance of each microbial taxon.

In summary, our study assessed the circadian rhythm of rumen microbes in terms of their population and composition under different feeding regimes and after rumen fluid transplantation. Compared to the previous studies that reported the diurnal oscillations of rumen microbiota in dairy cattle [15] and fecal microbiota in mice [16], we further classified the rhythmic taxa into FTR and MFR taxa. This approach has filled the knowledge gap in screening microbes that can be manipulated through feeding strategies. The identified consistent and inconsistent circadian rhythms of the microbial taxa after rumen transplantation indicated that not all microbes respond to exogenous microbes in the same way. The circadian rhythm of the microbes could be one of the regulatory mechanisms that make them more resilient or sensitive to interventions. With the specific responsive microbial information obtained in the present study, we can apply the most appropriate strategies to manipulate target taxa. Specifically, adjusting the feeding strategy should be used when targeting FTR taxa, while introducing exogenous microbes (e.g., probiotics) should target microbes that show responsiveness after rumen fluid transplantation. This knowledge can be adapted to other mammalian species for future microbiome manipulations to improve host functions. Beyond the scientific contributions of this study, there is an opportunity to enhance the education of future veterinarians, technicians, and farmers by emphasizing the practical applications of understanding microbial dynamics in the rumen. Recent literature on veterinary dairy cow nutrition education provides valuable insights into the best practices for preparing students to handle the complexities of modern dairy farm practice [86]. Our study provides a reference for strategies to manipulate the rumen microbiome by targeting specific taxa. This can help farmers adopt the best practices for manipulating the rumen microbiome to improve production.

## Conclusion

**Fig. 9:**
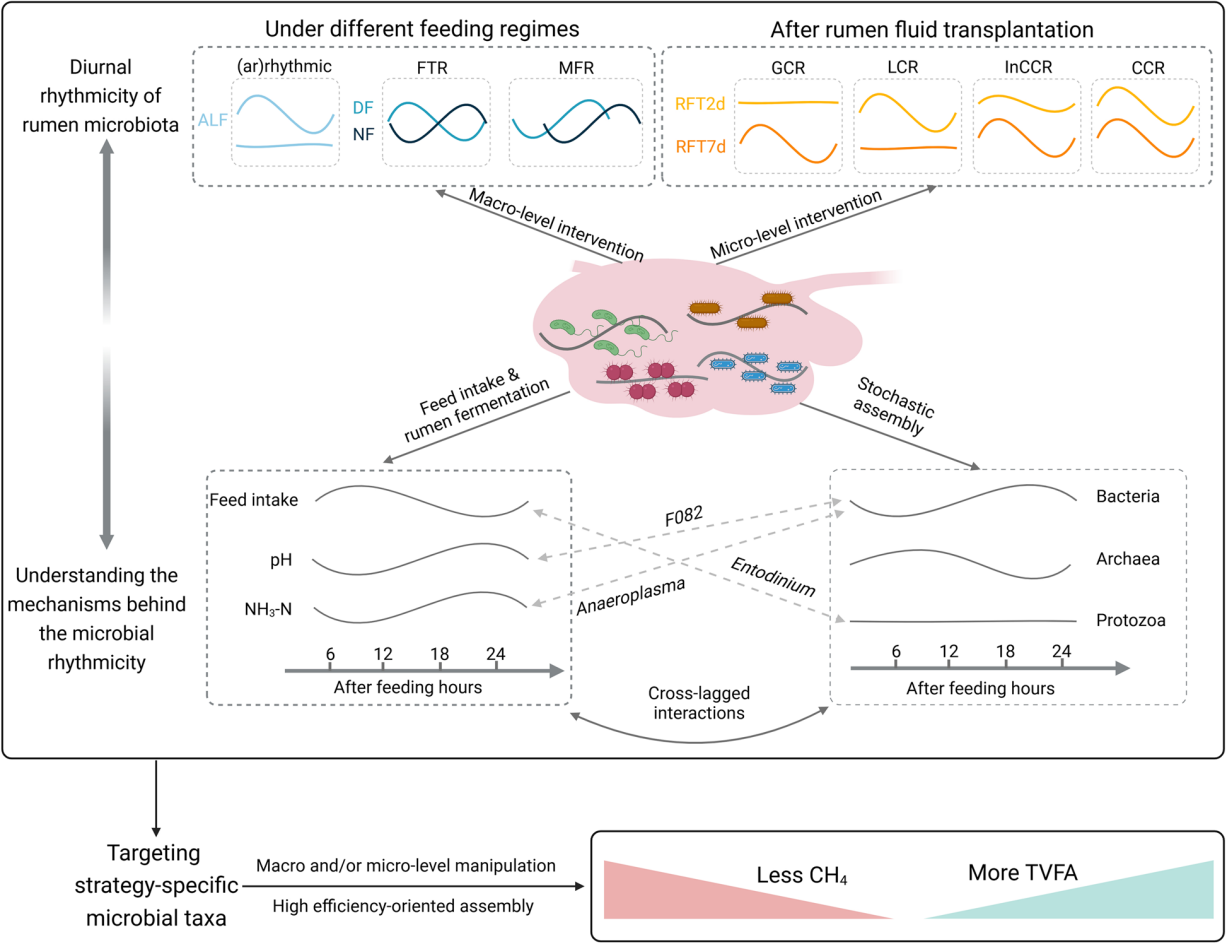
Summary of the study. ALF, ad libitum feeding; DF, daytime feeding; NF, nighttime feeding; RFT2d, within 48 h after rumen fluid transplantation; RFT7d, 7 days after RFT.[Aikira1] FTR, feeding time responsive taxa; MFR, multi-factor responsive taxa; GCR, gain circadian rhythm taxa; LCR, loss circadian rhythm taxa; CCR, consistent circadian rhythm taxa; InCCR, inconsistent circadian rhythm taxa; TVFA, total volatile fatty acids; NH_3_-N, ammonia nitrogen[Aikira2] [Aikira1]CE: This is unidentified paragraph from coast [Aikira2]CE: This is unidentified paragraph from coast

Our study revealed the circadian rhythm of rumen bacteria, archaea, and protozoa, and it also clarified how the different feeding regimes and introduction of exogenous microbiota affect these circadian rhythms (Fig. 9). The classified FTR, MFR, consistent, and inconsistent circadian rhythm of the relative abundance of microbial taxa highlighted the importance of strategies to target specific microbial taxa based their resilience to external factors when manipulating the rumen microbiota for improved feed efficiency and lower methane emissions in ruminants. Additionally, this knowledge can be adapted to other mammalian species for precise microbiome manipulations. However, future study with shorter sampling intervals is required to fully understand the daily dynamics of rumen microbiota. Meanwhile, the effect of animal well-being/stress response to the multi-time sampling within a day should be taken into account for the relationships with rumen microbial shifts. Although we processed the rumen fluid collected via esophageal tubing for microbial profiling, it may not represent the whole communities associated with feed particles, which may have varied circadian rhythms. Further studies should involve rumen-cannulated dairy cows to assess both solid-associated microbes and those in ruminal fluid. Finally, in-depth sequencing and follow-up experiments are also supposed to be conducted to further verify the current findings.

## Supplementary Information

Supplementary Material 1: Fig. S1. Hypothesized conceptual model of cross-lagged panel model

Supplementary Material 2: Fig. S2. Hypothesized conceptual model of partial least squares path modeling. TVFA: total volatile fatty acids, NH3-N: ammonia nitrogen, ECH4: estimated methane

Supplementary Material 3: Fig. S3. The circadian rhythm of rumen bacterial, archaeal, and protozoal alpha diversity (Chao1 and Shannon). ALF: ad libitum feeding; DF: daytime feeding; NF: nighttime feeding; RFT2d: within 48 h after rumen fluid transplantation; RFT7d: 7 days after RFT. The fitted curve indicated a significant circadian rhythm

Supplementary Material 4: Fig. S4. The circadian rhythm of ruminal bacteria, archaea, and protozoa population. ALF: ad libitum feeding; DF: daytime feeding; NF: nighttime feeding; RFT2d: within 48 h after rumen fluid transplantation; RFT7d: 7 days after RFT. The fitted curve indicated a significant circadian rhythm

Supplementary Material 5: Fig. S5. Ruminal bacterial, archaeal, and protozoal β diversity between the donor and recipient

Supplementary Material 6: Fig. S6. The cause-and-effect relationship among feed intake, ruminations, microbiota, and ruminal fermentation parameters. (A) Correlations among ruminal microbial diversity, microbiota population, fermentation profiles, feed intake, and rumination based on linear mixed model analysis. * *P* < 0.05,** *P* < 0.01, *** *P* < 0.001. The bacteria (B), archaea (C), and protozoa (D) were analyzed respectively. Observed variables are represented in a rectangular form, and the latent variables are represented in an elliptical form. Redline indicated a negative correlation and the blueline indicated a positive correlation. The line thickness indicated the coefficient value, and the significance of the coefficient was tested by bootstrap, * *P* < 0.05, ** *P* < 0.0.

Supplementary Material 7: Fig. S7. The comparation of the circadian rhythm patterns of microbial taxon *Moryella* between relative abundance (A) and estimated absolute abundance (B). (C) The difference in the peak time that calculated based on relative abundance and estimated absolute abundance for the rhythmic taxa. ALF: ad libitum feeding; DF: daytime feeding; NF: nighttime feeding; RFT2d: within 48 h after rumen fluid transplantation; RFT7d: 7 days after RFT. The fitted curve indicated a significant circadian rhythm

Supplementary Material 8: Suppl file S1. The ingredients and chemical composition of diet for the experiment dairy cows

Supplementary Material 9: Suppl file S2. The circadian rhythm information of ruminal microbial taxa

Supplementary Material 10: Suppl file S3. The circadian rhythm information of functional pathways in ruminal microbiota

Supplementary Material 11: Suppl file S4. The information of the top 20 bins that contribute to HoS and DL process, respectively. HoS. homogeneous selection, DL: dispersal limitation

Supplementary Material 12: Suppl file S5. The circadian rhythm information of feed intake, rumination time, and rumen fermentation parameters

Supplementary Material 13: Suppl file S6. The cross-lagged correlation between microbial taxa and feed intake, as well as rumination, and ruminal fermentation parameters

## Data Availability

All the amplicon sequencing data have been deposited into the NCBI Sequence Read Archive (SRA) under the accession number PRJNA1142764 (bacteria), PRJNA1142767 (archaea), and PRJNA1142908 (protozoa). No datasets were generated or analysed during the current study.

## Abbreviations

ALF: Ad libitum feeding
ASVs: Amplicon sequencing variants
CLPM: Cross-lagged panel model
DL: Dispersal limitation
DR: Drift and others
DF: Daytime feeding
ECH_4_: Estimated methane
FTR: Feeding time responsive
HD: Homogenizing dispersal
HeS: Heterogeneous selection
HoS: Homogeneous selection
iCAMP: Infer community assembly mechanisms by phylogenetic bin-based null model analysis
LMM: Linear mixed model
MFT: Multi-factor responsive
NH_3_-N: Ammonia nitrogen
NF: Nighttime feeding
PCR: Polymerase chain reaction
PLS-PM: Partial least squares path modeling
qPCR: Quantitative polymerase chain reaction
RFT: Rumen fluid transplantation
TVFA: Total volatile fatty acid
VFA: Volatile fatty acid
