# Supplementary figures and images for: Microbial diurnal rhythmicity in the rumen fluid impacted by feeding regimes and exogenous microbiome providing novel mechanisms regulating dynamics of the rumen microbiome
Source: Microbiome. 2025 Jun 16;13:142. doi: 10.1186/s40168-025-02134-6 (PMC12168421; doi:10.1186/s40168-025-02134-6)

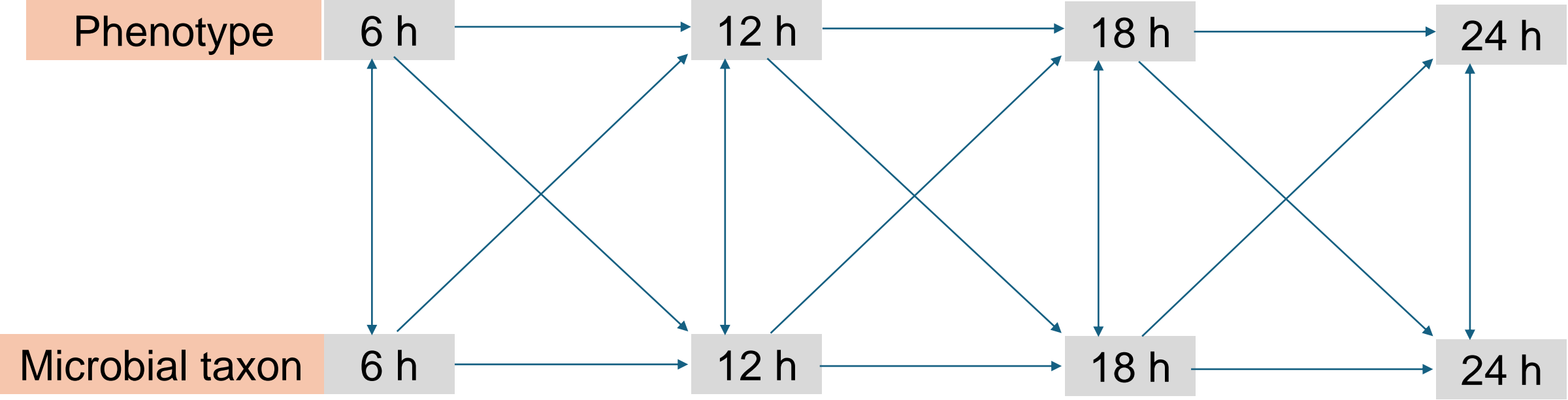

Supplement: Supplementary file 2 — Supplementary Material 1: Fig. S1. Hypothesized conceptual model of cross-lagged panel model [file 40168_2025_2134_MOESM1_ESM.pdf]

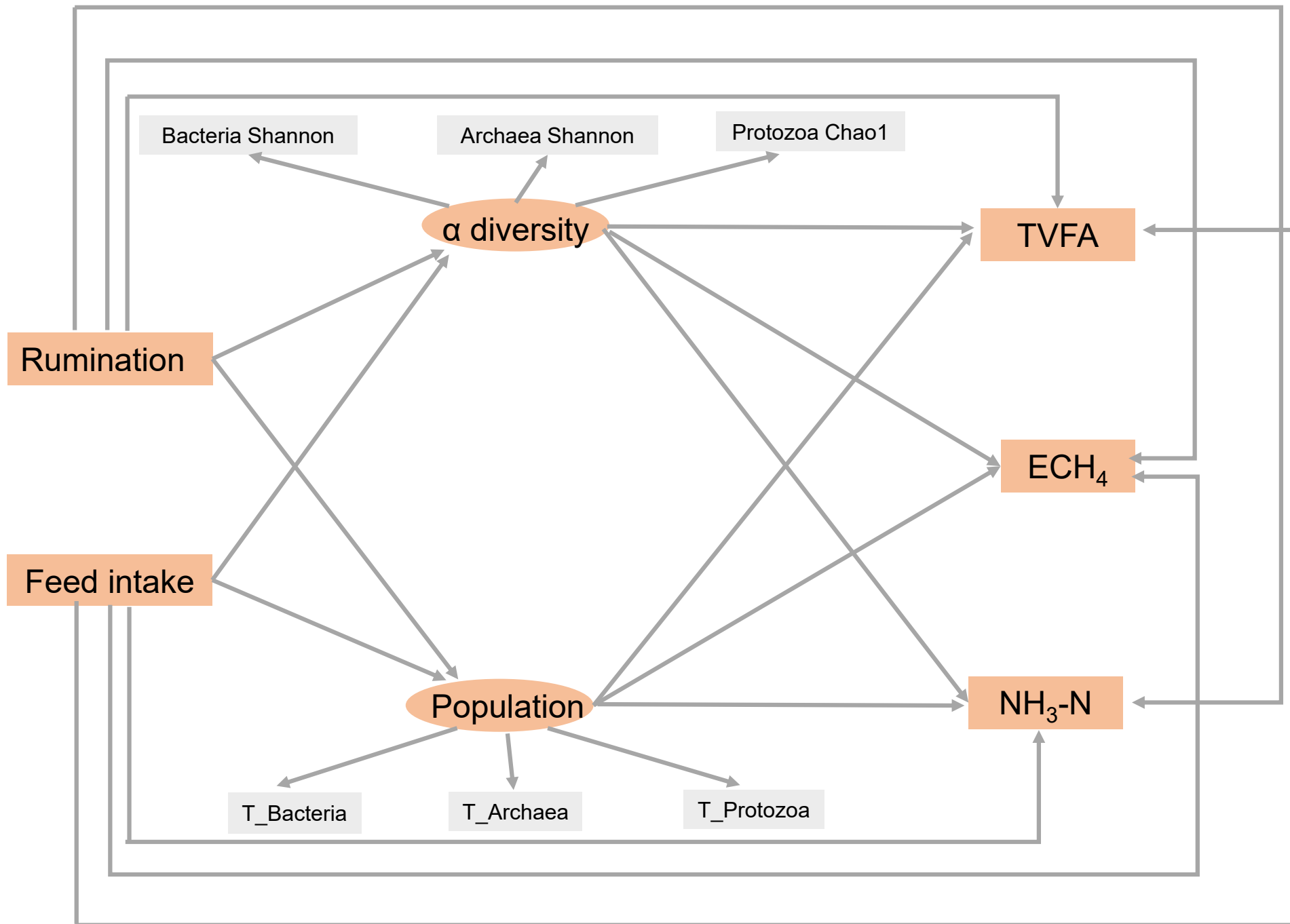

Supplement: Supplementary file 3 — Supplementary Material 2: Fig. S2. Hypothesized conceptual model of partial least squares path modeling. TVFA: total volatile fatty acids, NH3-N: ammonia nitrogen, ECH4: estimated methane [file 40168_2025_2134_MOESM2_ESM.pdf]

A

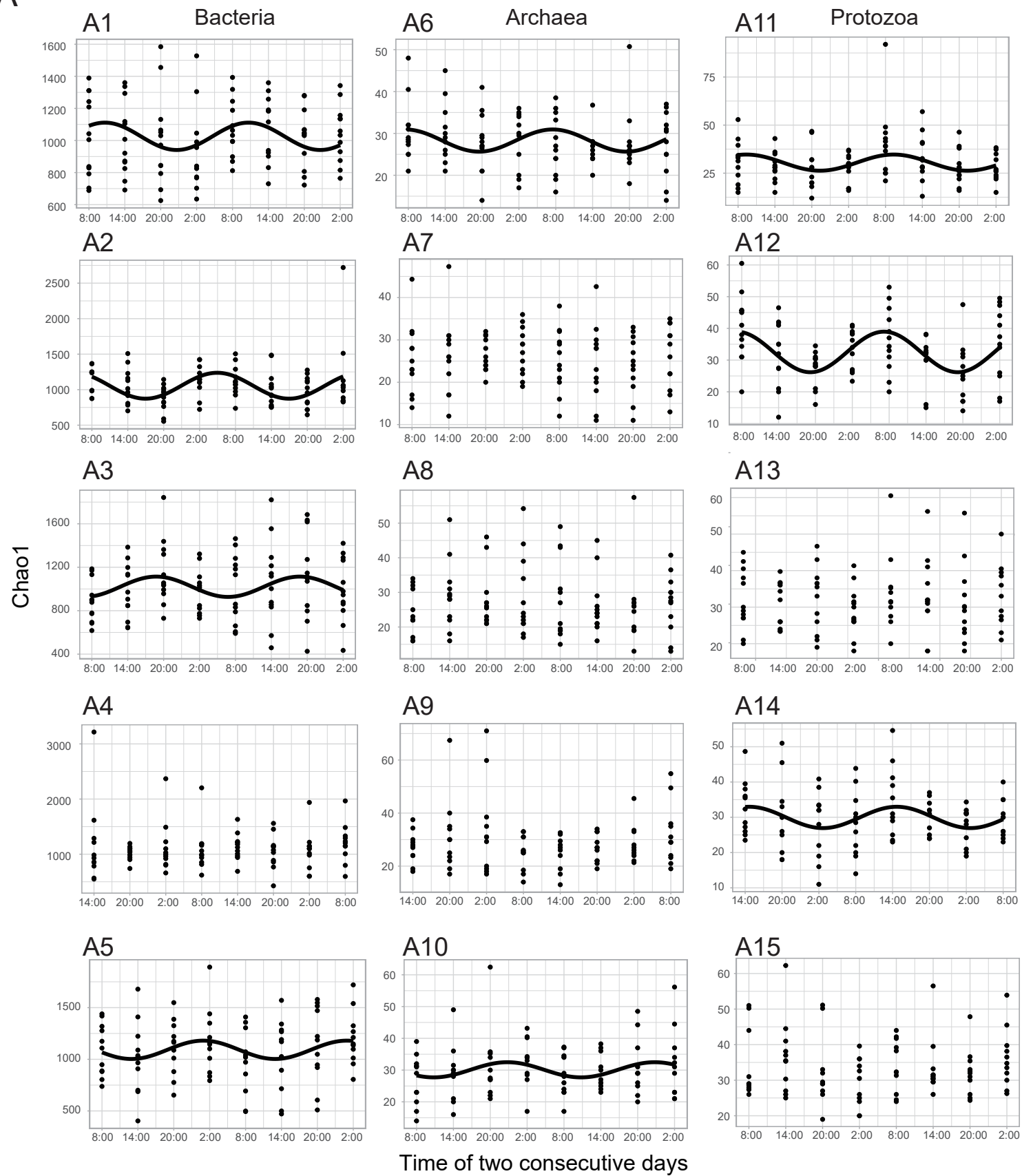

B

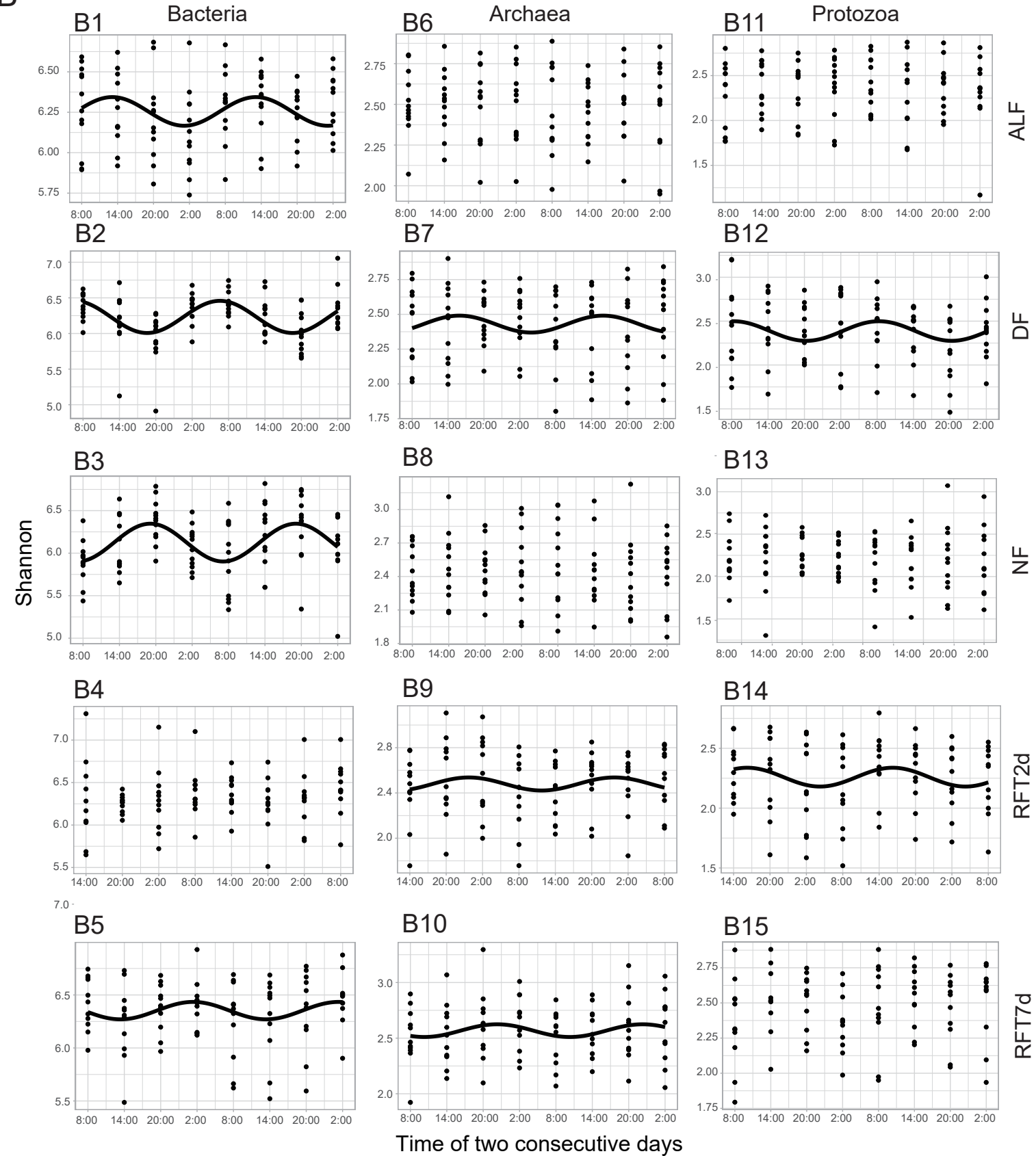

Supplement: Supplementary file 4 — Supplementary Material 3: Fig. S3. The circadian rhythm of rumen bacterial, archaeal, and protozoal alpha diversity (Chao1 and Shannon). ALF: ad libitum feeding; DF: daytime feeding; NF: nighttime feeding; RFT2d: within 48 h after rumen fluid transplantation; RFT7d: 7 days after RFT. The fitted curve indicated a significant circadian rhythm [file 40168_2025_2134_MOESM3_ESM.pdf]

Copy Numbers Log<sub>10</sub>/mL

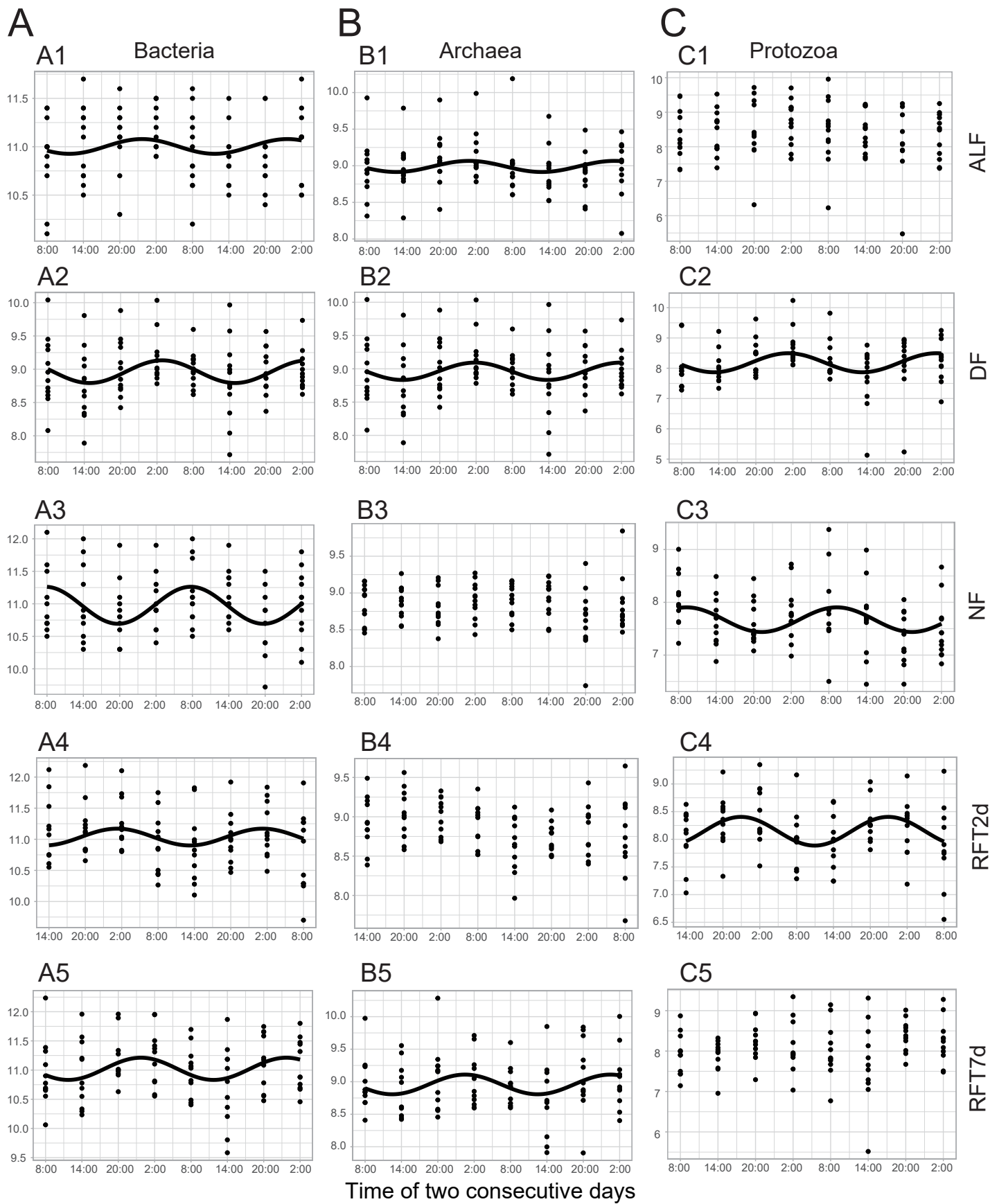

Supplement: Supplementary file 5 — Supplementary Material 4: Fig. S4. The circadian rhythm of ruminal bacteria, archaea, and protozoa population. ALF: ad libitum feeding; DF: daytime feeding; NF: nighttime feeding; RFT2d: within 48 h after rumen fluid transplantation; RFT7d: 7 days after RFT. The fitted curve indicated a significant circadian rhythm [file 40168_2025_2134_MOESM4_ESM.pdf]

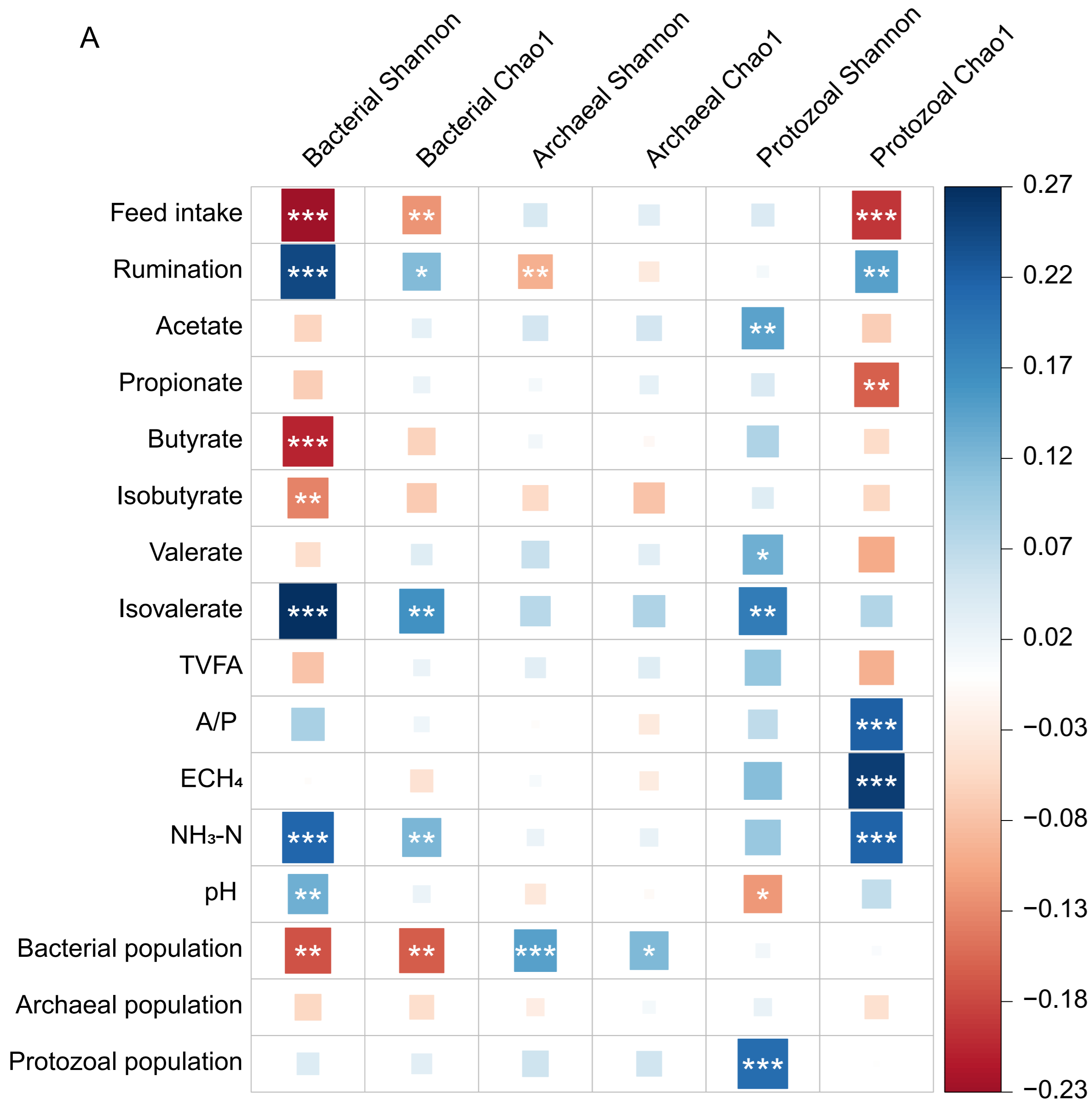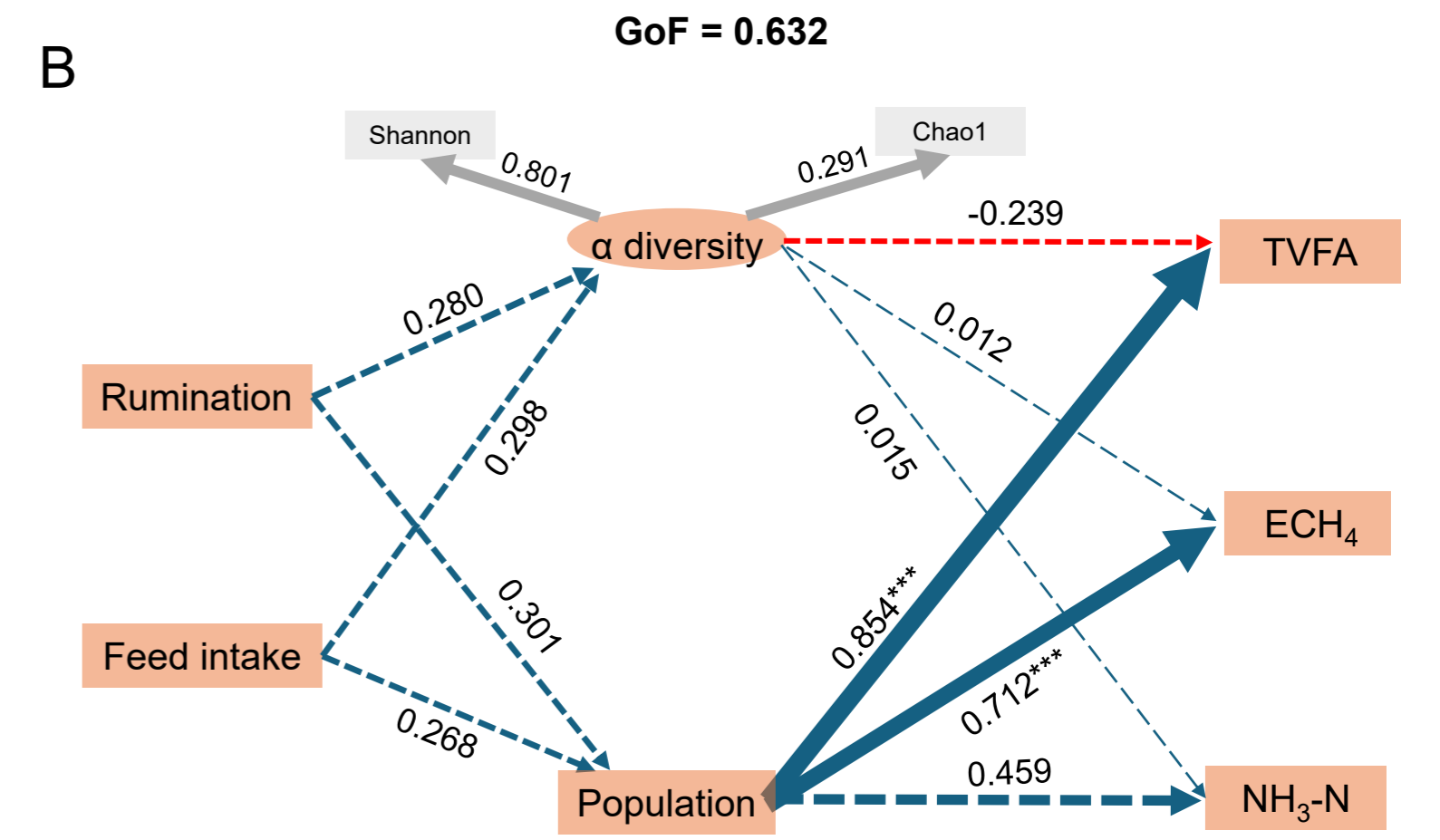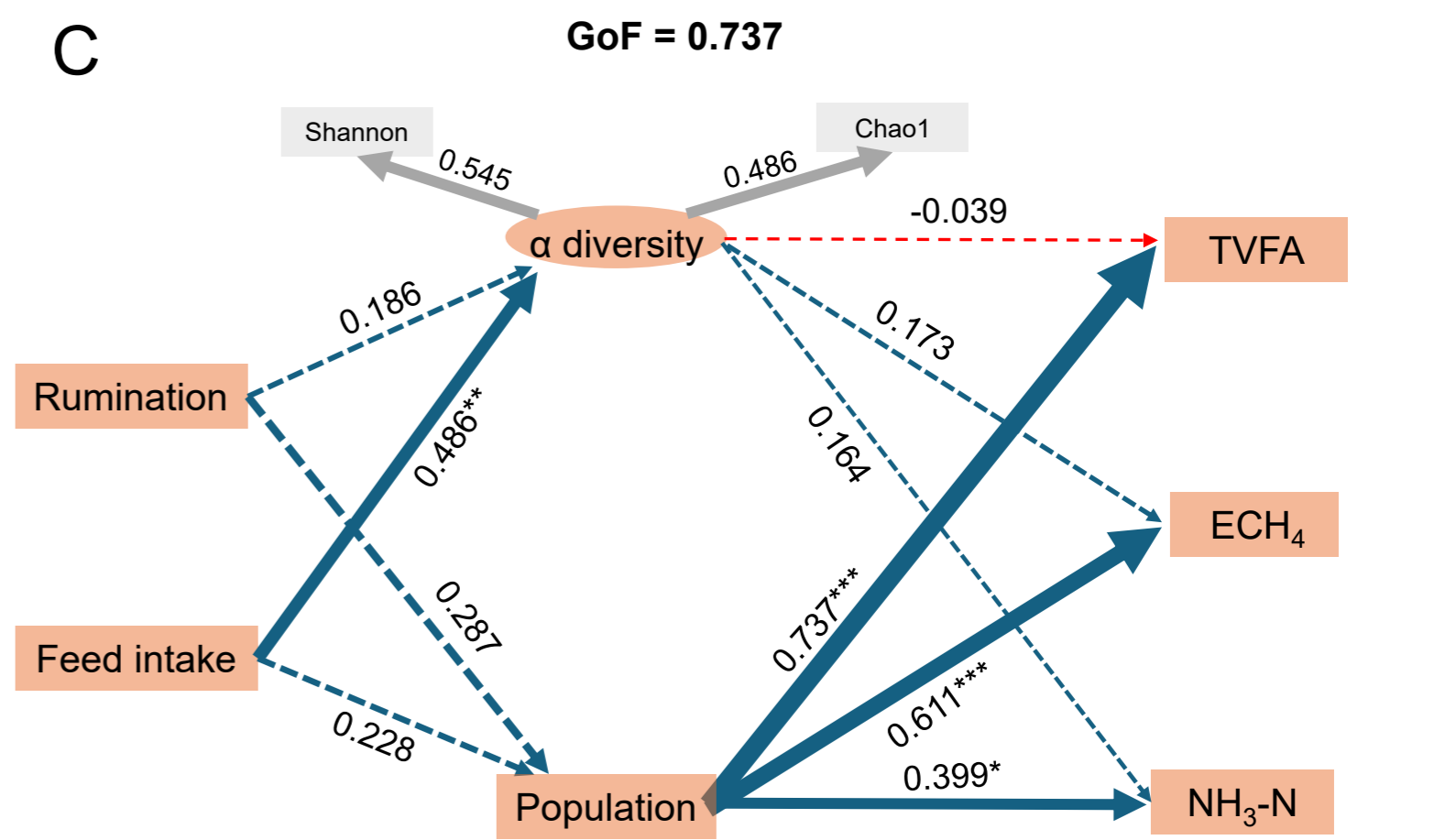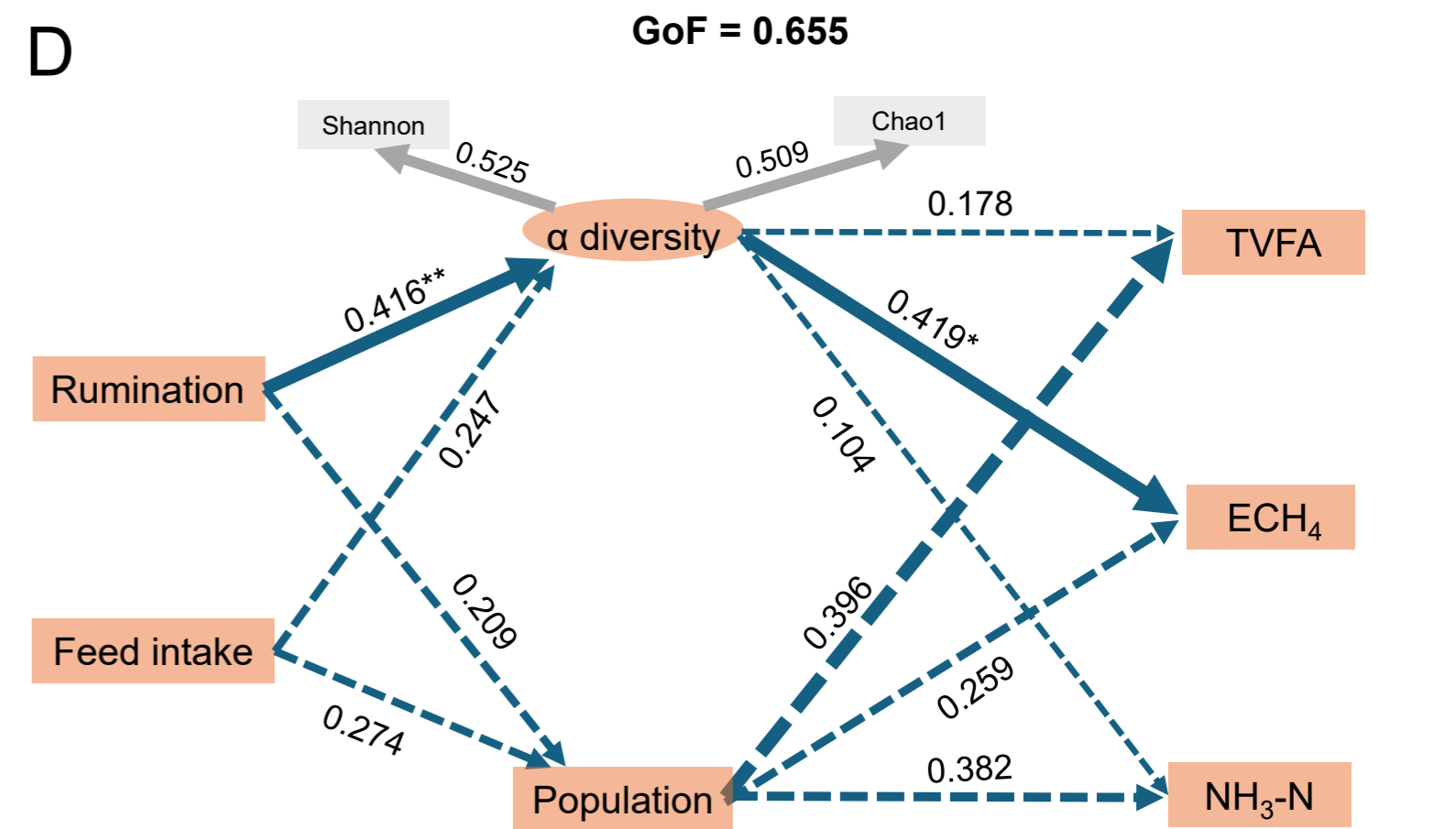

Supplement: Supplementary file 7 — Supplementary Material 6: Fig. S6. The cause-and-effect relationship among feed intake, ruminations, microbiota, and ruminal fermentation parameters. (A) Correlations among ruminal microbial diversity, microbiota population, fermentation profiles, feed intake, and rumination based on linear mixed model analysis. * P < 0.05,** P < 0.01, *** P < 0.001. The bacteria (B), archaea (C), and protozoa (D) were analyzed respectively. Observed variables are represented in a rectangular form, and the latent variables are represented in an elliptical form. Redline indicated a negative correlation and the blueline indicated a positive correlation. The line thickness indicated the coefficient value, and the significance of the coefficient was tested by bootstrap, * P < 0.05, ** P < 0.0. [file 40168_2025_2134_MOESM6_ESM.pdf]

A

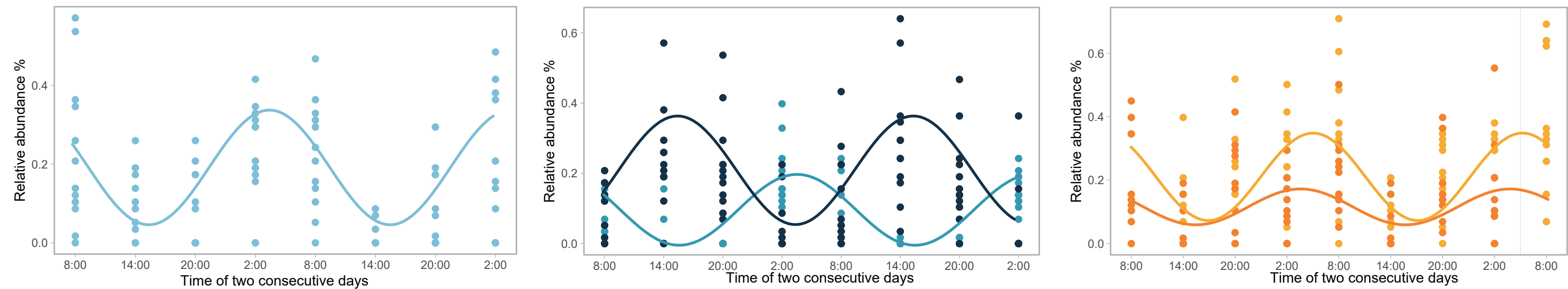

B

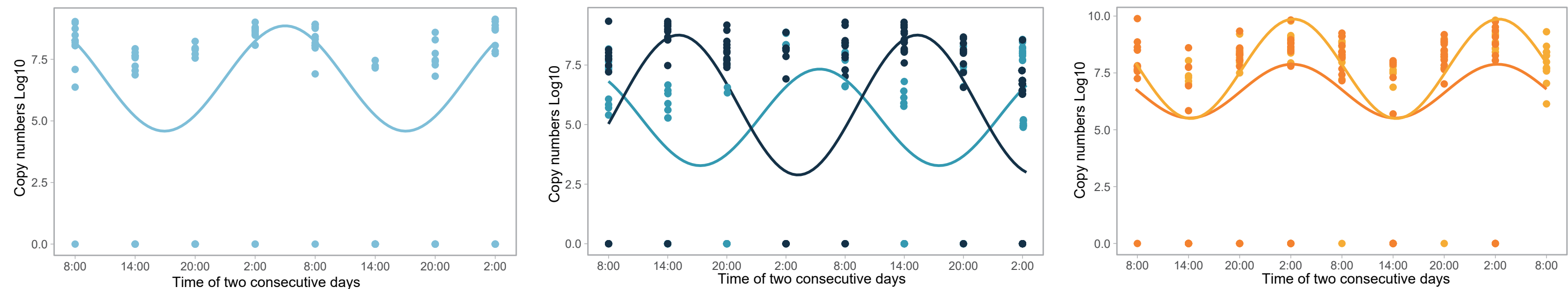

Groups

- ALF
- DF
- NF
- RFT2d
- RFT7d

C

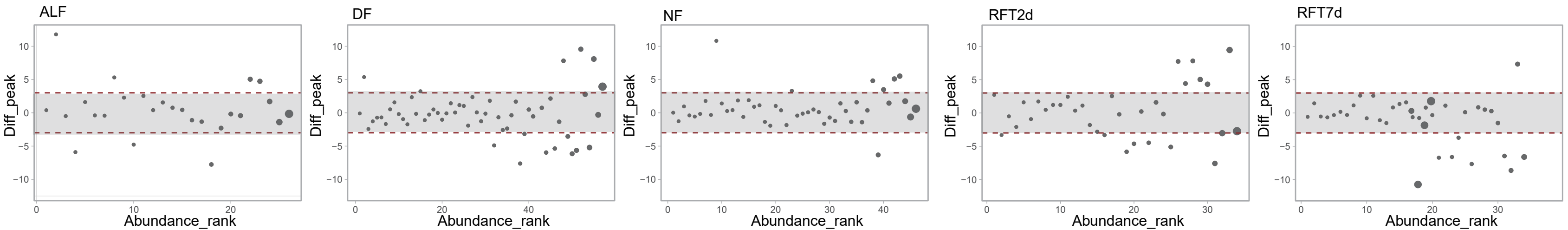

Supplement: Supplementary file 8 — Supplementary Material 7: Fig. S7. The comparation of the circadian rhythm patterns of microbial taxon Moryella between relative abundance (A) and estimated absolute abundance (B). (C) The difference in the peak time that calculated based on relative abundance and estimated absolute abundance for the rhythmic taxa. ALF: ad libitum feeding; DF: daytime feeding; NF: nighttime feeding; RFT2d: within 48 h after rumen fluid transplantation; RFT7d: 7 days after RFT. The fitted curve indicated a significant circadian rhythm [file 40168_2025_2134_MOESM7_ESM.pdf]
