# Supplementary material for: Microbial diurnal rhythmicity in the rumen fluid impacted by feeding regimes and exogenous microbiome providing novel mechanisms regulating dynamics of the rumen microbiome
Source: Microbiome. 2025 Jun 16;13:142. doi: 10.1186/s40168-025-02134-6 (PMC12168421; doi:10.1186/s40168-025-02134-6)
Supplement: Supplementary file 6 — Supplementary Material 5: Fig. S5. Ruminal bacterial, archaeal, and protozoal β diversity between the donor and recipient [file 40168_2025_2134_MOESM5_ESM.pdf]

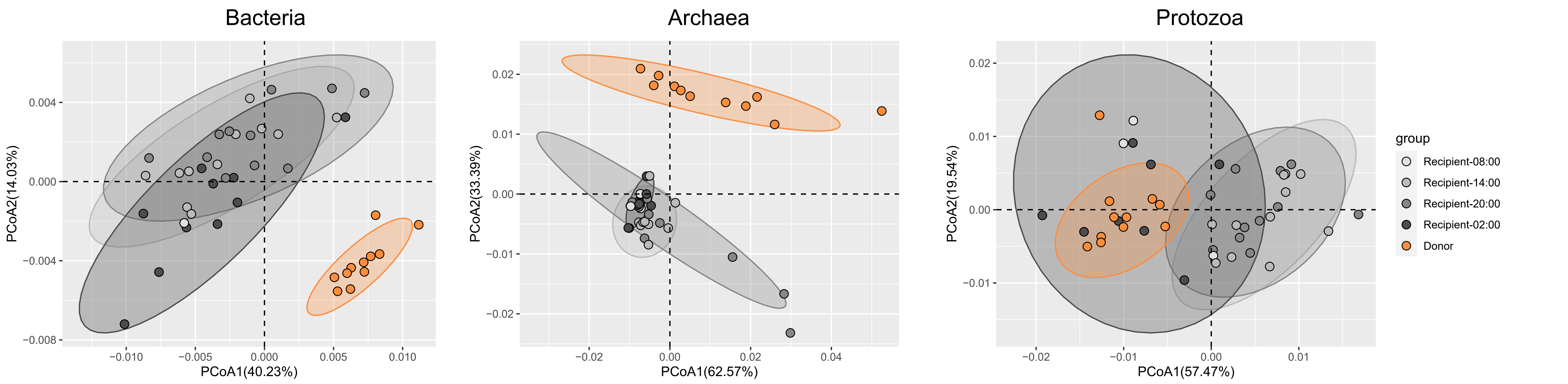

	P value
Donor vs Recipient-08:00	< 0.01
Donor vs Recipient-14:00	< 0.01
Donor vs Recipient-20:00	< 0.01
Donor vs Recipient-02:00	< 0.01

	P value
Donor vs Recipient-08:00	< 0.01
Donor vs Recipient-14:00	< 0.01
Donor vs Recipient-20:00	< 0.01
Donor vs Recipient-02:00	< 0.01

	P value
Donor vs Recipient-08:00	0.04
Donor vs Recipient-14:00	< 0.01
Donor vs Recipient-20:00	< 0.01
Donor vs Recipient-02:00	0.03
